# DNA Sequence Preferences of Transcriptional Activators Correlate More Strongly than Repressors with Nucleosomes

**DOI:** 10.1016/j.molcel.2012.06.028

**Published:** 2012-07-27

**Authors:** Varodom Charoensawan, Sarath Chandra Janga, Martha L. Bulyk, M. Madan Babu, Sarah A. Teichmann

**Affiliations:** 1 MRC Laboratory of Molecular Biology, Hills Road, Cambridge CB2 0QH, UK; 2 Department of Biochemistry, Faculty of Science, Mahidol University, Bangkok 10400, Thailand; 3 Integrative Computational BioScience (ICBS) Center, Mahidol University, Nakhon Pathom 73170, Thailand; 4 School of Informatics, Indiana University Purdue University, Indianapolis, IN 46202, USA; 5 Center for Computational Biology and Bioinformatics, Indiana University School of Medicine, Indianapolis, IN 46202, USA; 6 Division of Genetics, Department of Medicine, Brigham and Women's Hospital and Harvard Medical School, Boston, MA 02115, USA; 7 Department of Pathology, Brigham and Women's Hospital and Harvard Medical School, Boston, MA 02115, USA; 8 Harvard-MIT Division of Health Sciences and Technology, Harvard Medical School, Boston, MA 02115, USA

## Abstract

Transcription factors (TFs) and histone octamers are two abundant classes of DNA binding proteins that coordinate the transcriptional program in cells. Detailed studies of individual TFs have shown that TFs bind to nucleosome-occluded DNA sequences and induce nucleosome disruption/repositioning, while recent global studies suggest this is not the only mechanism used by all TFs. We have analyzed to what extent the intrinsic DNA binding preferences of TFs and histones play a role in determining nucleosome occupancy, in addition to nonintrinsic factors such as the enzymatic activity of chromatin remodelers. The majority of TFs in budding yeast have an intrinsic sequence preference overlapping with nucleosomal histones. TFs with intrinsic DNA binding properties highly correlated with those of histones tend to be associated with gene activation and might compete with histones to bind to genomic DNA. Consistent with this, we show that activators induce more nucleosome disruption upon transcriptional activation than repressors.

## Graphical abstract

**Graphical Abstract:**

**Figure:**
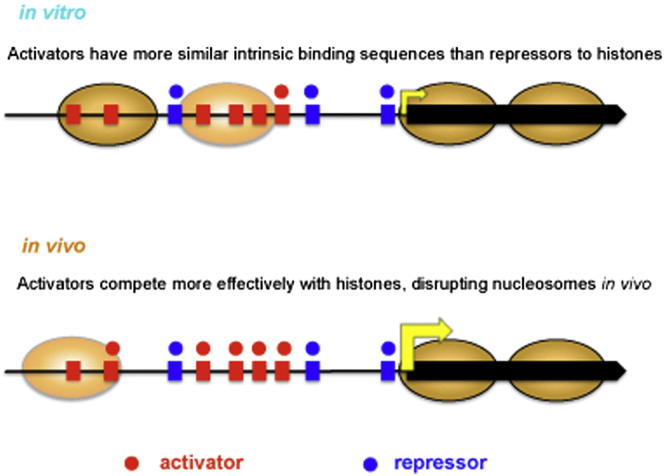

**Highlights:**

► We studied DNA-binding preferences for yeast nucleosomes and 137 TFs ► The majority of yeast TFs have intrinsic binding sequences similar to histones ► Activators have more similar intrinsic binding sequences than repressors to histones ► Activators compete more effectively with histones, disrupting nucleosomes formation

## Introduction

Transcription factors (TFs) provide specificity to the transcriptional machinery through the recognition of particular DNA sequences, enabling them to control expression of target genes. The fundamental mechanisms of transcriptional regulation are different between the two types of cellular organisms. In prokaryotes, the level of transcription largely depends on the binding strength of RNA polymerases and TFs to DNA (Wade et al., 2005). In eukaryotes, by contrast, DNA template accessibility is more restricted since genomic DNA is highly condensed, being bound by histone octamers and packed into higher order chromatin structures (Kornberg and Lorch, 1999).

Genomic DNA sequences occluded by nucleosomes are less accessible, which prevents TFs from freely interacting with their cognate sites on DNA due to steric hindrance (Struhl, 1999). Earlier studies have demonstrated the impact of nucleosome binding position on determining TF binding site (TFBS) accessibility (Field et al., 2009; Lee et al., 2007; Owen-Hughes and Workman, 1994; Segal et al., 2006; Yuan et al., 2005). Interestingly, Liu and coworkers (Liu et al., 2006) have shown that the computational prediction of in vivo binding of Leu3, a *Saccharomyces cerevisiae* TF, significantly improved when nucleosome occupancy was taken into account. In contrast, the prediction of sites bound by purified Leu3 in vitro did not improve, even though the binding motifs are indistinguishable in vivo and in vitro. This study underlined the global role of nucleosomes in determining the pattern of TF binding in living cells.

The intrinsic affinity of histones for DNA is by no means the only factor that influences the in vivo binding configuration of TFs and histones (Owen-Hughes and Workman, 1994). Numerous studies have shown that other DNA-binding proteins, including sequence-specific TFs and chromatin remodeling factors, can interact with nucleosome-occluded TFBSs and increase TF accessibility by disrupting, unwrapping, or repositioning nucleosomes upon transcriptional activation in vivo as well as in vitro (Bai et al., 2011; Fedor et al., 1988; Morse, 1993; Piña et al., 1990; Polach and Widom, 1995; Richard-Foy and Hager, 1987). Reciprocally to the work by Liu and coworkers, which showed that nucleosome occupancy improved in vivo TF binding prediction, Dai and colleagues (Dai et al., 2009) have demonstrated that the prediction of nucleosome positioning dynamics can be enhanced by integrating TF binding information.

Nucleosome and TF binding events are known to be interdependent; however, there is no consensus to what extent the binding events of the two types of proteins influence one another (Kaplan et al., 2009, 2010; Pugh, 2010; Tsankov et al., 2010; Zhang et al., 2009). Furthermore, there is no defined principle for how different TFs interact with nucleosomes to bring about specific regulatory outcomes (i.e., activation versus repression). Nucleosomes were conventionally associated with gene repression, and earlier detailed studies have shown for individual TFs that they can induce nucleosome repositioning upon transcriptional activation (Bai et al., 2011; Buck and Lieb, 2006; Ganapathi et al., 2011; Koerber et al., 2009; Yarragudi et al., 2004). Nevertheless, other studies have suggested that this is by no means the only generic rule (Shivaswamy et al., 2008; Wyrick et al., 1999; Zaugg and Luscombe, 2012).

Other open questions include how TFs access their cognate sites on genomic DNA preoccupied by nucleosomes and whether they tend to compete with histone octamers to bind to overlapping DNA sequence or simply choose different binding sites. A recent genome-scale study has demonstrated that the TF p53 preferentially binds to genomic regions with high intrinsic nucleosome occupancy in human (Lidor Nili et al., 2010). It remains to be seen whether this is also true for other TFs.

In this study, we have investigated whether the intrinsic DNA binding specificities of TFs tend to correlate with histone octamers in binding to DNA. We have addressed this question by systematically integrating and comparing several high-throughput data sets of binding specificities of nucleosomes and TFs in the budding yeast *S. cerevisiae*, a useful model of basic properties of transcriptional regulation in animals in the sense that ∼80% of fungal sequence-specific TF families and many histone-modifying enzymes are shared with metazoans (e.g., Charoensawan et al., 2010a, 2010b; Kim and Buratowski, 2009). Based on the intrinsic DNA-binding sequence preference of individual TFs and nucleosomal histones, we compute the number of TFs that share similar binding sites with histones on naked genomic DNA, and hence are likely to compete for overlapping sequences. We investigate how the intrinsic binding sequences of activating and repressing TFs overlap with those of nucleosomes, based on genome-wide in vitro occupancy profiles. Finally, we address how TFs of different regulatory modes (e.g., activators, repressors) influence in vivo nucleosome occupancy upon binding to their cognate sites in living cells.

## Results

### A Global Study of DNA-Binding Dynamics of TFs and Nucleosomes

**Figure 1:**
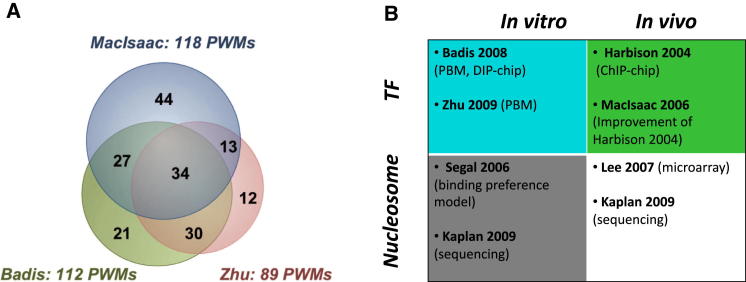
Summary of Data Sets Used in This Study (A) Venn diagram of the numbers of TFs, comprising DNA-binding data reported in three earlier studies (Harbison et al., 2004; Badis et al., 2008; Zhu et al., 2009). TF binding information is available (as PWMs) for 181 TFs altogether, while there are 201 PWMs from PBM experiments (Badis et al., 2008; Zhu et al., 2009), among 137 unique TFs. (B) Summary of all TF and nucleosome binding data sets used in this study. In vitro and in vivo TF binding preference data sets are highlighted in cyan and green, respectively. In vitro and in vivo nucleosome profiles are highlighted in gray and white, respectively. See also Figure S1 and Table S1.

**Figure 2:**
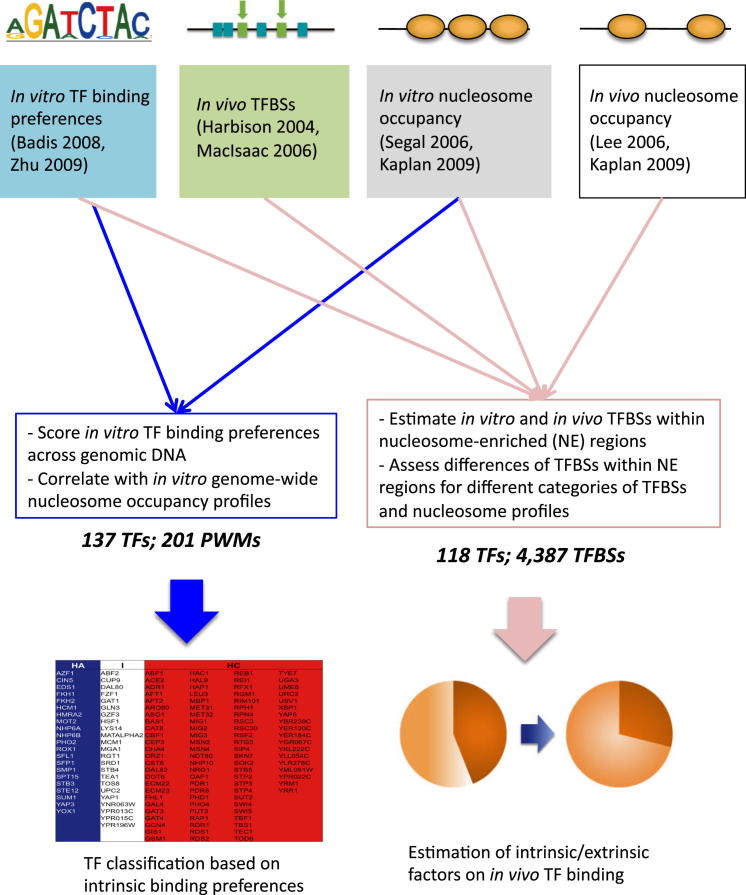
Summary of Analysis Methods Data sets used in this study can be divided into four groups: (1) in vitro TF binding preferences from PBM experiments (Badis et al., 2008; Zhu et al., 2009), (2) in vivo TF binding sites from ChIP-chip (Harbison et al., 2004) (MacIsaac et al., 2006), and genome-wide nucleosome occupancy profiles determined (3) in vitro and (4) in vivo (Kaplan et al., 2009; Lee et al., 2007; Segal et al., 2006). In vitro TF binding preferences were used to score against the entire budding yeast genomic DNA. The predicted genome-wide TF binding preference landscapes were individually correlated against genome-wide nucleosome occupancy profile. We classified TFs into histone-correlated (HC), intermediate (I), and histone-anticorrelated (HA) groups according to these correlation coefficients (Figure 3 and Table 1). All four types of data sets were combined to compute the fractions of predicted and in vivo TFBSs likely to be occluded by nucleosomes, based on occupancy profiles in vitro and in vivo (Figure 5). See also Figure S2 and Table S1.

To study global dynamics of TF and nucleosome binding, we have exploited comprehensive in vitro and in vivo data sets available for *S. cerevisiae*. The data sets used and the analyses performed in this study are summarized in Figures 1 and 2, respectively. In brief, in vitro sequence binding preferences were determined with purified TFs or histones, and custom-designed oligonucleotides or naked genomic DNA. Because the binding event depends on the sequence preference between TF (or histone octamer) and DNA, and is not influenced by other DNA-binding proteins (e.g., different TFs, histones), we regard this as the “intrinsic” DNA-binding preference. For TFs, intrinsic binding sequences were obtained from two large-scale protein binding microarray (PBM) studies (Badis et al., 2008; Zhu et al., 2009), where purified TFs were assayed for binding to custom-designed double-stranded DNA arrays (Berger et al., 2006) (201 position weight matrices [PWMs] among 137 unique TFs in total, Figure 1A). For nucleosomes, genome-wide in vitro nucleosome occupancy was determined from a probabilistic model representing the DNA sequences preferred by nucleosomal histones (Segal et al., 2006) and reconstitution of purified histones on naked genomic DNA (Kaplan et al., 2009). For the latter study, nucleosome-occluded sequences are less likely to be digested by micrococcal nuclease and were determined using next-generation sequencing (see the Supplemental Experimental Procedures).

The other type of data set contains the DNA binding sites of TFs and nucleosomes determined in vivo. These in vivo binding profiles capture the “overall” outcome of the combined effect from intrinsic (e.g., binding sequence preference) and extrinsic factors (e.g., competition or cooperation with other TFs, chromatin remodelers, binding of transcription initiation complex) that influence TF and nucleosome binding configuration in the cell. In vivo TFBSs used here are from ChIP-chip experiments, in conjunction with several evolutionarily conserved site searches (Harbison et al., 2004; MacIsaac et al., 2006). The in vivo nucleosome occupancy profiles used here were derived from yeast grown in YPD (rich medium), and other growth media (Kaplan et al., 2009; Lee et al., 2007). Extracted chromatin was treated with micrococcal nuclease, followed by sequencing-based identification of protected DNA. Table S1 (available online) summarizes the number of overlapping TFs from different high-throughput studies.

### The Majority of TFs Have an Intrinsic Binding Sequence Preference Similar to Histones

In eukaryotes, chromatin maintains the restrictive transcriptional ground state by blocking the binding of RNA polymerases and associated DNA-binding proteins. For TFs to bind to their cognate sites, the occluding nucleosomes have to be removed or the tightly wrapped DNA has to be at least partially unwound. Here, we report our investigation of the similarity between the genomic sequences intrinsically preferred by different TFs and by histones, by first assuming individual proteins can freely choose any genomic DNA sequence they prefer.

We used in vitro experimentally derived PWMs (Badis et al., 2008; Zhu et al., 2009) to score the entire yeast genome and assigned PWM scores to all possible binding sites, by moving the scoring window one base pair at a time (see Figures S1 and S2A for illustration). The PWM score assigned to each site represents the likelihood that the purified TF would bind to the site on naked DNA, and thus the intrinsic sequence preference between TF and DNA. We repeated this analysis for each of 201 PWMs (see a full list in Table S1) and correlated these genome-wide PWM scores (intrinsic binding likelihoods) of each TF individually to the in vitro nucleosome occupancy profiles from two studies (Kaplan et al., 2009; Segal et al., 2006) (Figures 1B and 2). For this analysis, we used in vitro nucleosome occupancy rather than nucleosome positioning profiles because the in vitro nucleosome occupancy data represent a quantitative measure of the intrinsic likelihood that each base pair of the yeast genomic DNA is occupied by nucleosomes (ranging from 0 to 1) (see discussion in Pugh, 2010). To quantify this correlation, we computed Pearson correlation coefficients between the intrinsic nucleosome occupancy and TF binding preference for each TF.

**Figure 3:**
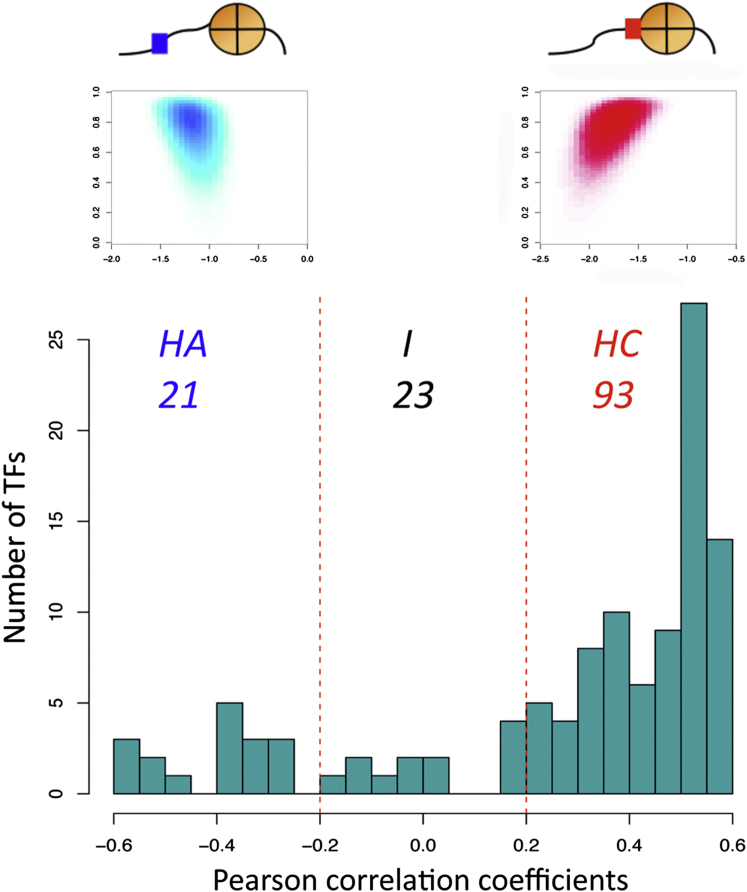
Histogram of Pearson Correlation Coefficients between Genome-wide Intrinsic DNA Binding Preferences of TFs and Nucleosomal Histones Out of 137 yeast TFs with available PWMs (Badis et al., 2008; Zhu et al., 2009), 93 TFs (∼70%) intrinsically prefer DNA binding sequences highly similar the regions also preferred by histones on naked DNA (i.e., histone-correlated group). The insets describe heatmaps correlating the genome-wide TF binding likelihoods of Rox1 (blue), and Abf1 (red), on the x axis, against the intrinsic nucleosome occupancy profiles on the y axis. The Pearson correlation coefficients between the two variables are −0.27 and 0.53, respectively, with the p values of linear model fitting < 2.2 × 10^−16^. See Figure S3A for high-resolution figures. See also Figure S3 and Tables S1, S2, and S3.

Both positive and negative correlations are observed. For example, the specific binding preference across the yeast genome of the TF Rox1 is negatively correlated with that of nucleosomes (blue heatmap in Figure 3 and Figure S3A), whereas that of Abf1 is positively correlated (red heatmap) (p values from linear model fitting < 2.2 × 10^−16^ for both TFs). We also checked for consistency using the Spearman correlation coefficient instead of Pearson (see also the Supplemental Experimental Procedures for details).

Based on these correlation coefficients, we categorized TFs into three groups: “histone correlated” (HC), “histone anticorrelated” (HA), and “intermediate” (I), using arbitrarily divided equal intervals that cover the entire range of the correlation coefficients (Figure 3 and Table 1). While the correlation coefficients can be binned in different ways, it is clear from the histogram that the majority of yeast TFs have overlapping binding sequences to those of histones across the genome. Using this criterion, about two-thirds of TFs that have PWMs available (93 out of 137, or ∼68%) have a binding sequence preference highly similar to that of histones, and thus fall into the HC group. These correlations are not found in random shuffling experiments of nucleosome occupancy profiles and are not an artifact of the information content and quality of the PWMs.

Since the intrinsic binding sequence preferences of the HC class TFs heavily overlap with those of histones, one would expect their in vivo TF binding sites, experimentally determined using high-throughput ChIP-chip, in conjunction with evolutionarily conserved site searches (Harbison et al., 2004; MacIsaac et al., 2006), to be occupied by nucleosomes more often than sites of the HA class TFs. Indeed, we find this to be true regardless of the nucleosome binding profiles used (Figures S4A–S4D).

### How Does Activator versus Repressor Binding Correlate with Nucleosomal Sequence Preference?

How do we explain the positive and negative correlations between intrinsic binding preferences of TFs and histones? The DNA-binding domains (DBDs) of TFs direct the proteins to their cognate sites and bind to those sites in a sequence-specific manner. Consequently, we first investigated the influence of DBD families on the correlations of TF and nucleosome binding preference (summarized in Table S3). We observed DBD families that tend to have positive (APSES, Gal4, HLH, SANT, zf-C2H2) or negative (Forkhead, HMG, Homeobox) correlations with nucleosome binding. In addition, we also noted that the binding specificity motifs of all TFs in the HC group contain significantly lesser A/T content than those of the HA group (∼0.37 versus ∼0.78, p value ∼10^−8^, Mann-Whitney). This is consistent with earlier studies showing that poly(dA-dT) stretches incorporate poorly into nucleosomes because of their relatively high rigidity (Nelson et al., 1987; Yuan et al., 2005).

**Figure 4:**
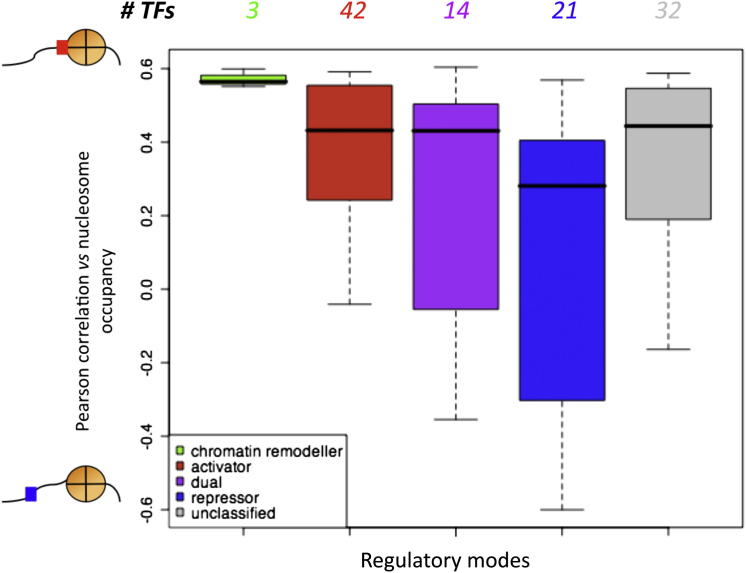
Activators Tend to Have Higher Correlation with Nucleosome Sequence Profiles than Repressors Box plots of the Pearson correlation coefficients between TF and nucleosome binding preferences, plotted separately for different regulatory modes (activator/repressor). Shown here as an example is the correlation between the binding preferences of 112 TFs (PWMs) from the Badis et al. (2008) data set against of the nucleosome occupancy profile from the Kaplan et al. (2009) data set. Numbers above the boxes indicate numbers of TFs in each category. The black bar in each box is the median correlation coefficient value, while the top edge of each box is the first quartile of the distribution, and the bottom edge the third quartile. The whiskers delimit the smallest and largest values of correlation coefficients of TFs for each regulatory mode group. Outliners are not shown. The average correlation with nucleosome binding preference profiles of activators is significantly higher than that of repressors (Mann-Whitney p value ∼0.02). See Figure S4E for the 89 PWMs from the (Zhu et al., 2009) data sets, p value ∼0.005. Please note that we focused on one PWM data set at a time for this plot due to the quantitative nature of the TF/nucleosome comparison. See also Figure S4 and Tables S2 and S3.

Next, we asked whether the TF regulatory modes (i.e., activation, repression, etc.) can be linked to the TF-histone correlation (i.e., HC, HA). Out of 137 TFs with available PWMs from PBM experiments (Badis et al., 2008; Zhu et al., 2009), 99 TFs (∼72%) have regulatory modes characterized. The regulatory modes are based on information in the *Saccharomyces* Genome Database (Dwight et al., 2002) with supporting experimental evidence, and on additional data from systematic fluorescent reporter assays characterizing the *S. cerevisiae* TFs (Sharon et al., 2012). Overall, we found that activators show significantly higher correlation with nucleosome sequence profiles on average than repressors (p value ∼0.02 for the (Badis et al., 2008) (Figure 4 for 112 PWMs) and ∼0.005 for the (Zhu et al., 2009) (Figure S4E for 89 PWMs) data sets, Mann-Whitney test; whereas TFs that can act as activator or repressor (dual regulators) showed intermediate correlation. Chromatin remodelers seem to have highly similar binding sequences to histones, but there are too few of them to draw firm conclusions. This suggests that repressors are intrinsically less likely to compete with histones, and thus they can access their cognate sites more directly than activators.

### To What Extent Do Intrinsic Sequence Specificities of Activators and Repressors Influence Their In Vivo Binding Positions Relative to Nucleosomes?

What is the difference between in vitro and in vivo nucleosomal occupancy profiles for activators and repressors? We have shown that activators tend to have an intrinsic DNA-binding sequence preference more similar to that of histone octamers, in contrast to repressors (Figures 4 and S4E). We thus hypothesized that activators compete with histones more effectively than repressors and at the same time are more capable of disrupting or repositioning nucleosomes in vivo. To test this, we assessed the overall outcome of activator and repressor binding on nucleosome occupancy in vivo as compared to that in vitro.

**Figure 5:**
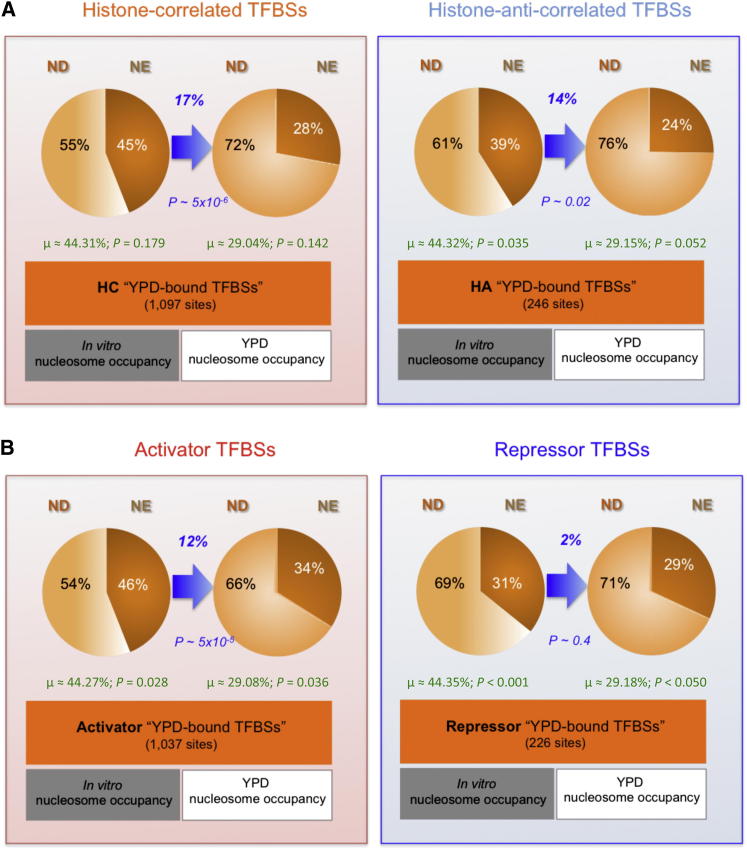
The Proportions of TF Binding Sites within Nucleosome-Enriched and Nucleosome-Depleted Regions The proportions of nucleosome-enriched (NE) and nucleosome-depleted (ND) TFBSs in YPD medium for HC and HA groups of TFs (A) and activators and repressors (B), based on in vitro and in vivo nucleosome occupancy profiles (Kaplan et al., 2009). Nucleosome-enriched proportions are shown in the darker shades. Focusing on the HC TFBSs in YPD, ∼55% were predicted to be nucleosome-depleted, based on the in vitro (intrinsic) nucleosome profiles. In contrast, with in vivo YPD nucleosome profiles, a significantly greater proportion of TFBSs were ND (∼17% difference, p value ∼5 × 10^−6^, Welch’s t test). For the HA TFBSs, the difference between the numbers of TFBSs occluded by the two nucleosome profiles (∼14%, p value ∼0.02) is less than that of the HC TFBSs. The expected averages from random shuffling experiments of nucleosome occupancies among all the YPD-bound sites, and the empirical p values that the actual values being greater or smaller than these averages are displayed in green text at the bottom of each pie chart. For activators and repressors, the difference between the numbers of nucleosome-enriched TFBSs according to in vitro and in vivo nucleosome profiles is considerably smaller for repressors (∼2%, p value ∼0.4) than activators (∼12%, p value ∼5 × 10^−5^). The fractions of nucleosome-enriched TFBSs of unclassified and other categories (neither activator nor repressor), and all other TFs combined are in Figure S5. See also Figure S5 and Table S2.

We focused on the binding sites occupied by TFs in YPD medium (Harbison et al., 2004; MacIsaac et al., 2006), which is the same medium used in the studies of in vivo nucleosome occupancy profiles (Kaplan et al., 2009; Segal et al., 2006). Following these authors’ analysis approach, we considered the sites with in vitro and in vivo nucleosome occupancies (Kaplan et al., 2009) greater than the genome-wide average to be nucleosome-enriched (NE), and nucleosome-depleted (ND) otherwise (see the Experimental Procedures). We computed the number of YPD-bound TFBSs within the NE and ND regions, for the HC versus HA TF groups (Figure 5A), for activators versus repressors (Figure 5B), as well as for total TFBSs of all categories (Figure S5).

We observe that roughly 45% of TFBSs bound by the HC TFs were predicted to be within the NE regions based on the in vitro nucleosome occupancy profiles, as shown in Figure S5A. This fraction is markedly greater than that of the sites bound by the HA TFs (∼39%). This is expected, however, because the HC/HA TFs were classified according to their intrinsic sequence preference against that of histones.

In order to compare TF and nucleosome occupancy under identical conditions in vivo, we switch from the nucleosome occupancy profile determined in vitro to the nucleosome profile obtained in vivo in the YPD medium (Kaplan et al., 2009). Now only ∼28% of these YPD-bound TFBSs were located in the in vivo NE regions, and thus ∼72% could be considered accessible by TFs (Figure 5A). The 17% (45%−28%) relative difference between nucleosome-enriched YPD-bound TFBSs according to the in vitro nucleosome profile and the profile derived in YPD is statistically significant (p value ∼5 × 10^−6^, Welch’s t test computed for the binding sites of different TFs) and is likely due to the influence of nonintrinsic factors such as in vivo TF binding, the recruitment of histone-modifying enzymes, and chromatin remodelers. We show in the Supplemental Experimental Procedures that this combined nonintrinsic effect of about 17% is greater than the effect of intrinsic histone-DNA binding preference on TF binding, consistent with earlier studies (Koerber et al., 2009; Owen-Hughes and Workman, 1994; Zhang et al., 2009). For the HA group, in contrast, the difference between the TFBSs within the in vitro and in vivo NE regions is smaller (∼14%) and less significant (p value ∼0.02).

**Figure 6:**
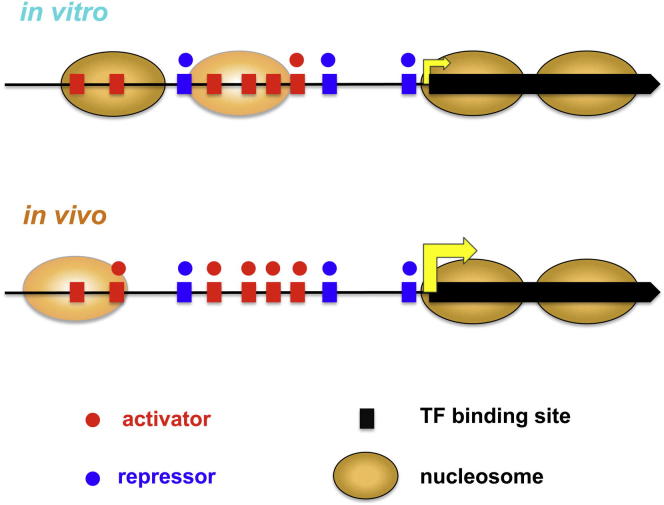
In Vitro and In Vivo TF and Nucleosome Binding Landscapes Based on intrinsic (in vitro) binding preferences, activators tend to have more similar DNA sequence preferences to those of nucleosomal histones, and their TFBSs might be less accessible, as compared with repressors. In yeast grown in YPD medium (in vivo), nucleosome occupancy around TFBSs of activators decreases dramatically, suggesting that activators are capable of outcompeting histones and accessing their binding sites during transcriptional activation, whereas repressors might synergize rather than compete with histones. See also Figure S6.

We observed a greater fraction of in vitro nucleosome-enriched TFBSs of activators when compared to those of repressors (∼46% versus ∼31%, Figure 5B and Table S2, sheet E), indicating that activators have more similar binding sites to the genomic regions intrinsically preferred by histones. This fraction of activator binding in the NE regions is greater than expected by chance, based on 1,000 shuffling experiments of nucleosome profiles, whereas that of repressors is lower than expected (green text in Figure 5, empirical p values of 0.028 and < 0.001, respectively). This is consistent with the result described earlier (Figure 4) that activators show similar intrinsic binding sequences to those of histones, whereas repressors have more different sequence preferences. Importantly, the fraction of TFBSs of activators within NE is markedly lower in vivo than in vitro, suggesting that activators are more capable of outcompeting histones and accessing their binding sites in living cells (∼12% reduction, p value ∼5 × 10^−5^). In contrast, there is no significant difference (∼2%, p value ∼0.4) between the nucleosome-enrichment at the TFBSs of repressors (Figure 5B, right panel). This indicates that repressors might synergize rather than compete with histones during transcriptional repression. The stabilization of chromatin, potentially preventing RNA PolII access to its template, may thus represent an important mechanism for transcriptional repression, as illustrated graphically in Figure 6.

In the above analysis, we have considered differences between DNA-binding in vitro versus in vivo of cells grown in YPD. What changes in TF and nucleosome binding occur across different in vivo yeast growth conditions? Systematic analysis of dynamics of TF and histone binding based on high-throughput data sets is possible for the nucleosome-correlated transcriptional activator Gal4 TF because the in vivo TFBSs (Harbison et al., 2004) and nucleosome occupancy profiles (Kaplan et al., 2009) are available for both YPD and galactose-supplemented media. According to the ChIP-chip data, Gal4 binds to eight promoters and regulates eleven target genes in total in both media (Figure S6). With the exception of the TFBS in the *GAL80* promoter, an inhibitor of Gal4 activity, all other binding sites switch to lower nucleosome occupancy upon galactose induction, and Gal4 activates these target genes. This result supports the model that TFs whose DNA binding is correlated with nucleosomes tend to coincide with nucleosome repositioning upon transcriptional activation.

In summary, comparison of in vitro and in vivo data sets of TF and nucleosome binding supports a model of nucleosome repositioning upon transcriptional activation, while nucleosomes tend to be more static upon transcriptional repression. Globally, this is consistent with activating TFs sharing intrinsic sequence specificity with nucleosomes, and repressors having more different sequence specificities than those of nucleosomes.

## Discussion

We used *S. cerevisiae* to elucidate the interdependent binding of TFs and nucleosomal histones in eukaryotes, because of the wealth of binding specificity data available for this organism. We have shown that the majority of yeast TFs have an intrinsic binding sequence preference that is positively correlated with that of nucleosomal histones (HC) (Figure 3). The enrichment in the HC TFs might be the result of coevolution of TFs and nucleosomes. While the formation of nucleosomes helps to minimize nonspecific DNA binding, the HC TFs are capable of displacing nucleosomes when the TFs are present at higher concentration, such as in response to environmental changes. This may add robustness to transcriptional regulation, and thus might be selected in evolution. In contrast, the HA group would be expected to be able to bind more directly to their cognate sites, which are more accessible in the context of chromatin.

The intrinsic binding preference of nucleosomes is thought to influence their genome-wide binding (Kaplan et al., 2009, 2010; Segal et al., 2006). However, recent studies have shown that other nonintrinsic factors, including TF binding, histone modification, and chromatin remodeling events, are at least as important as the intrinsic sequence preference on in vivo nucleosome organization (Bai et al., 2011; Koerber et al., 2009; Tsankov et al., 2010; Zhang et al., 2009). According to our independent investigation, we observed a significant reduction in nucleosome occupancy around experimentally determined TF binding sites, when we compared nucleosome profiles obtained in vitro versus in vivo. This confirms earlier findings that nonintrinsic factors have a significant impact on in vivo nucleosome occupancy around TF binding sites. Intriguingly, the most dramatic decrease of nucleosome occupancy was observed at the binding sites of the HC group as compared with the HA group, and activators as compared with repressors.

Our results indicate that activators might compete more effectively with histones to bind to similar DNA sequences, as compared with repressors. Thus, when activators bind to their cognate sites in vivo, this results in disruption, unwinding, or repositioning of nucleosomes (Figures 4 and 5B) and thus allows other TFs and the transcription initiation complex to bind to these regions and initiate transcription. Our results predict that sites bound by repressors should be more directly accessible, as their binding sites are less similar to those of nucleosomes. This may be more favorable for transcriptional repression rather than activation, as the cost to the cell of erroneous activation is greater than that of repression. Importantly, our results suggest the model that repressors might act at least in part by stabilizing transcriptionally repressive chromatin, rather than competing with nucleosomes (Figure 6).

Another important piece of evidence supporting the role of TF binding on nucleosome dynamics comes from analysis of data from two different environmental conditions. Considering the classic Gal4 model (a HC TF in our classification) (Bryant and Ptashne, 2003; Floer et al., 2010), we further investigated nucleosome occupancy in YPD and galactose-supplemented media, at the promoters of all known Gal4 target genes. We find that nucleosome occupancy around the Gal4 binding sites decreases upon galactose induction and leads to transcriptional activation. Another classic example is the activation of *PHO5* by phosphate deprivation. Pho4 (a HC TF) can compete with nucleosome formation and is essential for disruption of nucleosomes within promoters, thus allowing other proteins including Pho2 (a HA TF) to access the regions (Ertel et al., 2010; Lam et al., 2008; Svaren et al., 1994). Several other studies have experimentally demonstrated the ability of individual HC TFs to bind DNA and disrupt promoter nucleosomes in vivo, including Abf1, Rap1, Reb1, and Rsc3 (Bai et al., 2011; Buck and Lieb, 2006; Ganapathi et al., 2011; Hartley and Madhani, 2009; Koerber et al., 2009; Shivaswamy et al., 2008). Strikingly, Lickwar and coworkers (Lickwar et al., 2012) have recently demonstrated the direct competition between Rap1 and histones in budding yeast. They have shown that stable binding of Rap1 with high-affinity sites associates with long residence time on cognate sites and pronounced nucleosome depletion. A similar phenomenon where TFs displace histones to bind to overlapping sites on DNA upon gene activation is also observed in multicellular eukaryotes, for individual TFs or target genes (Ercan et al., 2011; Kumar et al., 2012; Tillo et al., 2010).

The results presented here link together studies on many individual TFs into an overall model, through our identification of their common correlation with histone recognition sequences. We achieved this by systematically analyzing the binding preferences of all known budding yeast TFs based on a single set of consistent criteria. This approach can serve as a platform for similar genome-wide analyses in higher eukaryotes.

Apart from providing insights into the global interplay between TF binding and nucleosome occupancy, our study also raises a number of interesting mechanistic questions. For instance, how do HC TFs and activators disrupt nucleosomes? At high concentrations, some TFs can directly displace and prevent histone binding at the TF cognate sites by steric hindrance (Polach and Widom, 1995). Alternatively, some TFs can indirectly disrupt nucleosomes by recruiting histone-modifying enzymes and ATP-dependent chromatin remodeling complexes (Buck and Lieb, 2006; Whitehouse et al., 2007), which have been shown to have a significant influence on gene expression variability (Choi and Kim, 2008). However, direct and indirect chromatin modifier-TF interactions cannot be easily distinguished (Steinfeld et al., 2007).

In addition, the location and configuration of TFBSs are also thought to facilitate the binding of TFs to DNA. Some TFs cooperatively bind to several binding sites within proximity. This can result in increased TF-DNA binding affinity and specificity (Bilu and Barkai, 2005; Hochschild and Ptashne, 1986), minimized nonfunctional binding (Wunderlich and Mirny, 2009), and decreased nucleosome occupancy (Miller and Widom, 2003; Wasson and Hartemink, 2009) and may fine-tune the expression level of target genes (Lam et al., 2008). It remains to be seen how different classes of TFBSs are organized in the context of binding sequence preference. For instance, two or more HC TFs may bind to closely co-occurring sites and thus cooperatively evict histones.

Our results also generate a direct testable prediction that increased repressor concentrations should cause less change in nucleosome organization than increased activator concentrations. This TF-nucleosome competition assay and other systematic experimental analyses on the interplay between different DNA-binding proteins will lead to a better understanding of the rules that govern the dynamics of transcriptional regulation under different environmental conditions.

## Experimental Procedures

Supplemental Experimental Procedures are available online.

### Large-Scale Transcription Factor and Nucleosome Binding Data Sets

We obtained 4,387 experimentally verified “in vivo TFBSs” (for 118 unique TFs) of *Saccharomyces cerevisiae* from MacIsaac et al. (2006). The in vitro DNA-binding specificity data were taken from two large-scale PBM studies (Badis et al., 2008; Zhu et al., 2009). A summary of all TFs used in this study with their gene names according to the SGD database (Dwight et al., 2002) is in Table S1. The regulatory mode information (activator/repressor) was also obtained from the SGD database. Only TFs that have experimentally supported evidence were classified as activator, repressor, dual regulator, or chromatin remodeler (A, R, D, and C, respectively). Additional activator/repressor information was obtained from systematic fluorescent reporter assays characterizing the *S. cerevisiae* TFs in Sharon et al. (2012). All other TFs were classified as unknown (U).

Genome-wide nucleosome occupancy data sets were obtained from three different studies: (1) nucleosome binding likelihoods based on a probabilistic model that represents the DNA sequences preferred by nucleosomal histones (Segal et al., 2006), (2) genome-wide in vivo nucleosome occupancy/positioning for yeast grown in YPD (rich) medium (Lee et al., 2007), and (3) genome-wide nucleosome occupancy profiles measured in vivo, and in vitro using naked DNA (Kaplan et al., 2009).

### Assigning TF Binding Likelihoods to Yeast Genomic Sequence

We scored the PWMs taken from two independent in vitro high-throughput PBM experiments (Badis et al., 2008; Zhu et al., 2009) against the *S. cerevisiae* genome from the SGD database (Dwight et al., 2002). We used Matrix-scan, available as part of the RSAT tools (Thomas-Chollier et al., 2008), to compute the “weight of sequence segment,” as described in (Hertz and Stormo, 1999), for all possible binding sequences in the yeast genome. The assigned PWM scores, which represent intrinsic TF binding likelihoods, were calibrated against the available DIP-chip data at 32 bp resolution for five TFs (Badis et al., 2008), and thus we used this 32 bp window average to represent the intrinsic binding likelihood for the rest of our analysis (see the Supplemental Experimental Procedures for more details).

### Correlating In Vitro TF Binding Preferences with In Vitro Nucleosome Binding Preference

We quantitatively assessed the similarities between the intrinsic DNA-binding preferences of TFs with nucleosomal histones by individually correlating the in vitro TF binding likelihoods (i.e., PWM scores) of all possible binding sequences in the entire yeast genome, to the genome-wide nucleosome occupancy profiles determined in vitro. The correlations were performed between the 32 bp means of TF binding likelihoods obtained by scoring PWMs (Badis et al., 2008; Zhu et al., 2009) as explained above, and the 32 bp means of intrinsic nucleosome occupancies (Kaplan et al., 2009; Segal et al., 2006). We then computed Pearson correlation coefficients between the computed intrinsic binding likelihood profiles of all TFs and the two independent in vitro nucleosome occupancy profiles (Kaplan et al., 2009; Segal et al., 2006). The distributions of correlation coefficients were divided into three equal intervals: HA, I, and HC. We also checked for consistency using the Spearman correlation coefficient and different averaging window sizes (see the Supplemental Experimental Procedures for details).

### Identifying TF Binding Sites Bound in YPD

Of the total 4,387 “in vivo TFBSs,” we identified those bound in YPD (rich medium) within 700 bp upstream of the translation start site. We used a p value threshold of 0.001 for the intergenic probes corresponding to the TFBSs in the ChIP-chip experiments of yeast grown in YPD medium (Harbison et al., 2004). Using these criteria, we identified 1,963 “YPD-bound TFBSs.” The TFBSs bound in galactose-supplemented medium were identified in the same manner.

### Estimating the Fractions of Binding Sites within Nucleosome-Enriched Regions

We superimposed the two in vitro genome-wide nucleosome occupancy profiles (Kaplan et al., 2009; Segal et al., 2006) onto the in vivo TFBSs and YPD TFBSs. For the Segal et al. (2006) data set, we considered the sites with nucleosome occupancy greater than the cutoff of 0.5 (very stable nucleosomes) as the NE sites, i.e., likely to be occluded by “stable” nucleosomes, in the same manner as the original publication. In contrast, the sites with nucleosome occupancy of less than 0.5, are considered to be ND sites. For the Lee et al. (2007) data set, we considered the sites within “well-positioned” and “fuzzy” nucleosomes as nucleosome enriched. For the Kaplan et al. (2009) data set, we also followed the authors’ initial analysis by defining the sites that have log ratios between the number of reads that cover a particular base pair and the average across the genome above zero, i.e., nucleosome occupancy above genome-wide average, as NE sites (and thus below zero as ND sites). The expected numbers of TFBSs within the NE and ND regions were the means of 1,000 iterations of randomly shuffling nucleosome occupancy profiles over the YPD-bound sites.

## Figures and Tables

**Table 1:** The Histone-Correlated and Histone-Anticorrelated TF Groups

HA	I	HC			
Azf1	Abf2	Abf1	Hac1	Reb1	Tye7
Cin5	Cup9	Ace2	Hal9	Rei1	Uga3
Eds1	Dal80	Adr1	Hap1	Rfx1	Ume6
Fkh1	Fzf1	Aft1	Leu3	Rgm1	Urc2
Fkh2	Gat1	Aft2	Mbp1	Rim101	Usv1
Hcm1	Gln3	Aro80	Met31	Rph1	Xbp1
Hmra2	Gzf3	Asg1	Met32	Rpn4	Yap6
Mot2	Hsf1	Bas1	Mig1	Rsc3	Ybr239c
Nhp6a	Lys14	Cat8	Mig2	Rsc30	Yer130c
Nhp6b	Matapha2	Cbf1	Mig3	Rsf2	Yer184c
Pho2	Mcm1	Cep3	Msn2	Rtg3	Ygr067c
Rox1	Mga1	Cha4	Msn4	Sip4	Ykl222c
Sfl1	Rgt1	Crz1	Ndt80	Skn7	Yll054c
Sfp1	Srd1	Cst6	Nhp10	Sok2	Ylr278c
Smp1	Stb4	Dal82	Nrg1	Stb5	Yml081w
Spt15	Tea1	Dot6	Oaf1	Stp2	Ypr022c
Stb3	Tos8	Ecm22	Pdr1	Stp3	Yrm1
Ste12	Upc2	Ecm23	Pdr8	Stp4	Yrr1
Sum1	Yap1	Fhl1	Phd1	Sut2	
Yap3	Ynr063w	Gal4	Pho4	Swi4	
Yox1	Ypr013c	Gat3	Put3	Swi5	
	Ypr015c	Gat4	Rap1	Tbf1	
	Ypr196w	Gcn4	Rdr1	Tbs1	
		Gis1	Rds1	Tec1	
		Gsm1	Rds2	Tod6	

The yeast TFs with available PWMs (Badis et al., 2008; Zhu et al., 2009) were analyzed to determine whether their intrinsic binding preferences are positively or negatively correlated to the intrinsic binding preference of nucleosomal histones (Kaplan et al., 2009; Segal et al., 2006). The TFs with weak correlation or disagreement between PBM or nucleosome binding preference publications were classified into an intermediate class. The correlation coefficients of all PWMs can be found in Table S1.

## Supplemental Information

Table S1. Table Summarizing all TFs Analyzed in This Study, Related to Figures 1, 2, and 3 and Table 1 The table includes the information on the latest common and standard gene names according to the SGD database, DNA-binding domains, TF domain architectures, lengths, AT content and Shannon entropy of PWMs, correlations between genome-wide binding preference of TF and nucleosome occupancy using average and maximal values to represent the 32 bp windows (for different TF and nucleosome data sets), regulatory mode (activator/repressor) and their evidence, and genomic locations. Header descriptions can be found in the second spreadsheet in the same file.

Table S2. Intrinsic TF Binding Likelihoods across the Yeast Genome and Number of Nucleosome-Enriched TF Binding Sites, Related to Figures 3, 4, and 5 Sheet A: For TFs with more than 50 binding sites (MacIsaac et al., 2006), the table provides the information on total number of sites, average intrinsic binding likelihoods assigned to all 32 bp sequences in the yeast genome, based on PWMs obtained from PBM studies (Badis et al., 2008; Zhu et al., 2009), in intergenic regions, and at the in vivo binding sites. Sheet B: Total numbers of TFBSs and those locating in nucleosome-enriched regions, based on different nucleosome occupancy data sets. For TFs with more than 50 binding sites (MacIsaac et al., 2006), the table provides the information on total number of sites for each TF, average binding likelihoods assigned to sites, numbers, fractions of sites occluded by nucleosomes based on different nucleosome binding profile, and the percentages of putative sites actually utilized by TFs in vivo. Sheet C: Same as in sheet A but sorted by HC/HA TF classes. Sheet D: Same as in sheet A but sorted by regulatory modes (activator/repressor). Sheet E: Total numbers of in vivo TFBSs and those locating in nucleosome-enriched (NE) regions, based on in vitro and in vivo nucleosome occupancy profiles, sorted by TFs and regulatory modes (activator/repressor).

Table S3. TF PWM Classification, Related to Figures 3 and 4 Sheet A: TF PWM classification, based on the intrinsic binding preference to DNA along the yeast genome (highlighted by DNA-binding family). Sheet B: TF PWM classification, based on the intrinsic binding preference to DNA along the yeast genome (highlighted by activator/repressor category).

Document S1. Supplemental Experimental Procedures and Figures S1–S6
