# Supplementary material for: DNA Sequence Preferences of Transcriptional Activators Correlate More Strongly than Repressors with Nucleosomes
Source: Mol Cell. 2012 Jul 27;47(2):183–92. doi: 10.1016/j.molcel.2012.06.028 (PMC3566590; doi:10.1016/j.molcel.2012.06.028)
Supplement: Document S1. Supplemental Experimental Procedures and Figures S1–S6 [file mmc4.pdf]

Molecular Cell, ***Volume 47***

Supplemental Information

DNA Sequence Preferences of Transcriptional Activators Correlate More Strongly than Repressors with Nucleosomes

Varodom Charoensawan, Sarath Chandra Janga, Martha L. Bulyk, M. Madan Babu, and Sarah A. Teichmann

Supplemental Experimental Procedures

Large-scale transcription factor and nucleosome binding data sets

We first obtained experimentally verified transcription factor binding sites (TFBSs) of budding yeast *Saccharomyces cerevisiae* (MacIsaac et al., 2006). These were determined using the high-throughput ChIP-chip (Chromatin immunoprecipitation with microarray detection) assays performed in different growth conditions (Harbison et al., 2004), in conjunction with several conservation-based motif discovery computational algorithms. We termed these experimentally determined TFBSs (in all growth conditions combined): "*in vivo* TFBSs". Due to the insufficient resolution of microarrays at the time, it was not possible to pinpoint where each TFBS located using the ChIP-chip experiment alone. Therefore, evolutionary conservation together with motif searches were used to help identify DNA sequences in intergenic regions. The motifs were generally conserved across closely related yeast species, and likely to have regulatory functions (MacIsaac et al., 2006). In our study, we used the *in vivo* TFBSs identified in (MacIsaac et al., 2006) at the most stringent criteria for both microarray binding (*P*-value cut-off 0.001), and conservation level available. This resulted in 4,387 high-confidence *in vivo* TFBSs of 118 unique TFs.

The *in vitro* DNA-binding specificity data was taken from two large-scale protein binding microarray (PBM) studies (Badis et al., 2008; Zhu et al., 2009), where the protein-DNA binding specificities were determined using purified TFs and customarily designed oligonucleotide sequences (Berger et al., 2006). We summarised the numbers of overlapping TFs among these three publications in Table S1 and Figure 1 in main text. The *in vivo* (MacIsaac et al., 2006) and *in vitro* (Badis et al., 2008; Zhu et al., 2009) DNA binding preferences from the above studies are available as Position Weight Matrices (PWMs).

A summary of all TFs used in this study with their gene names according to the SGD database (Dwight et al., 2002) can be found in Table S1. The table also provides the information on the DNA-binding domains (DBDs) and TF domain architectures obtained from the DBD database (Wilson et al., 2008). We also calculated the length and Shannon entropy (information content) for each PWM, obtained from the two PBM studies.

The regulatory mode information (activator/repressor) was obtained from the SGD database. Only TFs that have manually annotated GO with experimentally supporting evidence were classified as activator, repressor, dual regulator, or chromatin remodeller (A, R, D, C, respectively). Additional activator/repressor information was obtained from (Sharon et al., 2012). The rest were classified as unknown (U).

How complete is this combined TF dataset, compared with the predicted yeast TF repertoire? To assess this, we compared this combined set of TFs with available PWMs, with the list of TFs annotated computationally based on the presence of manually curated DNA-binding domains (DBDs), obtained from the DBD database (Wilson et al., 2008). We found

that the majority (~84%) of TFs that contain known DBDs have PWM information available. However, we noted that 33 TFs had identified binding motifs but were not annotated in the DBD database. These TFs might contain uncharacterised DBDs (PWM found *in vivo* and *in vitro, e.g.* Abf1 and Sum1) or might be co-binding proteins, which do not bind directly to DNA but through protein-protein interactions with other TFs instead (PWM found *in vivo* but not *in vitro, e.g.* Dig1 and Snf1). More discussion comparing the direct *versus* indirect TF-DNA interactions can be found in an earlier study (Gordan et al., 2009; Gordan et al., 2011). Nonetheless, as the combined TF binding specificity data set used here covers the majority (~84%) of the predicted yeast TF repertoire, we expect the insights gained from this analysis to be representative of all yeast TFs. The complete list of TFs, their DBDs and other protein domains contained, can be found in Table S1.

Genome-wide nucleosome occupancy data sets were obtained from three different studies:

(1) Nucleosome binding likelihoods based on a probabilistic model that represents the DNA sequences preferred by nucleosomal histones (Segal et al., 2006). In this study, genome-wide nucleosome occupancy was computationally predicted based on the intrinsic histone-DNA specificity. Thus, we regard this as an *in vitro* nucleosome profile.

(2) Genome-wide *in vivo* nucleosome occupancy/positioning for yeast grown in YPD (rich) medium (Lee et al., 2007), which were experimentally determined using a microarray at 4-bp resolution.

(3) Genome-wide nucleosome occupancy profiles measured *in vivo* in different growth conditions, as well as *in vitro* using naked DNA. The nucleosome-enriched DNA sequences were determined using parallel sequencing (Kaplan et al., 2009). In this study, the DNA sequences protected by nucleosomes from being digested by micrococcal nuclease (MNase) were determined using Illumina/Solexa sequencing, for yeast grown in the YPD, as well as galactose and ethanol supplemented media. For the *in vitro* nucleosome profile, histone octamers purified from chicken erythrocytes were reconstituted on yeast genomic DNA by salt gradient dialysis. We note that the biological relevance of this artificially reconstituted nucleosome profile is subjected to the conditions used in the salt dialysis (*e.g.* histone and DNA concentrations, pH, temperature). Nonetheless, it serves as a good representation for the intrinsic binding specificity of histone octamer and DNA, importantly because no other DNA-binding protein was present. The average nucleosome occupancy at nearly every base pair was calculated for each nucleosome profile in the same way, by dividing the number of reads (obtained from deep sequencing) that cover that base pair by the average number of reads per base pair across the genome. A summary of the data sets used in this study can be found in Figure 1 in main text and the rationale for using *in vivo* and *in vitro* data sets for certain analyses are explained in the next section.

Rationale for using in vitro and in vivo DNA-binding sequence preferences and binding position data sets

There are two types of sequence preference/binding position data used in our study: *in vitro* and *in vivo* (as summarised in Figure 1 and Figure 2).

In vitro data describe the "intrinsic" DNA-binding preferences of TFs and histones, excluding the effects of the competition/cooperation between the two kinds of proteins, and other DNA-binding proteins. For instance, *in vitro* binding preference of a TF was determined using purified TF and naked DNA, *e.g.* (Badis et al., 2008; Zhu et al., 2009). Thus, the TF can, in theory, bind to any genomic DNA sequence based on its own sequence preference, independent of the binding preferences of other TFs and histones. Similarly, *in vitro* nucleosome occupancy was discovered by allowing purified histone octamer to bind to naked

genomic DNA, without the presence of TFs and other DNA-binding proteins, *e.g.* in (Kaplan et al., 2009).

In contrast, *in vivo* data capture the "overall" outcome of the interaction between different TFs and histones in nuclei, at a given time and condition, in a cell population. Thus, the ultimate binding outcomes observed (*i.e.* bound genomic locations) of a particular TF or a histone octamer are influenced by the intrinsic binding preference of the molecule itself, as well as the non-intrinsic factors such as the binding specificities of all other proteins present in nuclei.

Assigning TF binding likelihood to yeast genomic sequence

We scored the PWMs taken from two independent *in vitro* high-throughput PBM experiments (Badis et al., 2008; Zhu et al., 2009) against the *S. cerevisiae* genome downloaded from the SGD database (May 2009). We used Matrix-scan, available as part of the RSAT tools (Thomas-Chollier et al., 2008), to compute the "weight of sequence segment", as described in (Hertz and Stormo, 1999), for all possible binding sequences in the yeast genome. This was carried out by moving a single base pair at a time, from the start to the end of each chromosome. By assuming that the computed PWM score represents the likelihood that DNA sequence is bound by TF on naked DNA, the TF binding likelihood is greatest when the DNA sequence is identical to the consensus motif. Taking the centre of each possible binding sequence to represent the position of that putative binding site, we assigned this "intrinsic TF binding likelihood" to all possible binding sequences across the yeast genome.

Benchmarking the predicted intrinsic binding likelihood obtained from PBMs with DIP-chip experiments

We used the PWMs determined by the PBM experiments to represent the intrinsic binding specificities between TFs and naked DNA, as described in the earlier section. At the same time, we are aware that using binding specificity determined by other methods such as DIP-chip (DNA immunoprecipitation with microarray detection) might be more biologically relevant compared with that obtained from PBM, mainly because genomic DNA is used instead of custom-designed oligonucleotide sequences (Liu et al., 2005). However, the lack of availability of DIP-chip data makes it unfeasible to use this for most TFs. We compared the DNA-binding profiles obtained from these two different methods by cross-validating the TF binding likelihoods assigned to the yeast genome, based on scoring the PWMs from PBMs (as described above), against the highly enriched TF-bound sequences determined using the DIP-chip experiments for the five TFs available (Leu3, Pho2, Pho4, Rap1, Rox1) (Badis et al., 2008). In the DIP-chip study, the TF-bound sequences were discovered using a 32-bp resolution microarray. As a result, we used the mean of PBM-derived PWM scores within 32-bp windows along the genome to compare against the highly enriched TF-bound sequences from the DIP-chip experiments. A flowchart summarising this benchmarking/correlating procedure can be found in Figure S1.

To assess the correlation between the DIP-chip highly enriched sequences and the top PBM-derived PWM score regions, we arbitrarily identified the top 5% intensity of the DIP-chip probes as TF-bound sequences. Then, we asked whether the PBM-derived PWM scores could distinguish the bound from non-bound sequences, according to the DIP-chip result. For all five TFs from the Badis and colleagues study (Badis et al., 2008), and four from the Zhu and colleagues study (Zhu et al., 2009) (as Rox1 was not available), the TF-bound sequences based on the DIP-chip experiments have significantly greater PWM scores than the non-bound sequences (P-values $< 10^{-15}$, Mann-Whitney test). We concluded that the high PBM-derived PWM scores correlated well with the DIP-chip highly enriched TF-bound sequences at 32-bp resolution, and thus used this 32-bp window average for the rest of our analysis. Using the mean PWM scores to represent 32-bp windows has an advantage over using the

maximal score, because it allows the contribution from both putative strong and weak binding sites (sites with high or low PWM scores) within the 32-bp windows to be taken into account.

Exhaustive search of putative binding sites

We assigned PWM scores (*i.e.* intrinsic TF binding likelihoods) to all possible binding sequences in the genome as explained earlier, and superimposed the positions of TFBSs experimentally verified by ChIP-chip experiments (MacIsaac et al., 2006), that is, *in vivo* TFBSs. We then assigned the mean PWM score of a 32-bp window to a binding site if the centre of that site falls within that 32-bp window (as example of Leu3 in Figure S2A). As one would expect, we observed that the PWM scores assigned to the *in vivo* TFBSs (green) are always higher than the intergenic regions (grey). This difference is highly significant in 32 out of the 38 PWMs that pass our criteria (P-value < 0.001, Mann-Whitney). In other words, functional TFBSs have greater intrinsic binding affinity (*i.e.* PWM scores) than the intergenic background.

For each TF, we defined the minimal PWM score assigned to any *in vivo* TFBSs determined by ChIP-chip as a minimal PWM score threshold. Next, we used this minimal intrinsic binding likelihood as a cut-off to search computationally for other 32-bp window in the intergenic regions with the PWM scores greater or equal to this cut-off, and terms them "putative TFBSs". In theory, we expect these putative TFBSs to be bound by the TF on naked DNA, if the TF-DNA interaction was based on the intrinsic sequence preference alone, and no extrinsic factors such as competition with other TFs and histones were involved.

What fraction of predicted in vitro TFBSs are utilised in vivo?

Eukaryotic DNA-binding motifs are known to be highly degenerate and thus match many genomic DNA sites, but only a small fraction of these putative binding sites are utilised *in vivo.* Can we estimate this fraction? Based on the intrinsic sequence preference of each TF for DNA, we computed the total number of putative TFBSs throughout the yeast genome, and calculated the fraction of these putative TFBSs, which are likely to be bound by TFs *in vivo*.

We first obtained the high-confidence "*in vivo* TFBSs" from a study by MacIsaac and colleagues (MacIsaac et al., 2006), using the most stringent cut-offs (4,387 sites), and the *in vitro* TF-binding sequence specificity from two PBM studies (Badis et al., 2008; Zhu et al., 2009). For those TFs with more than 50 TFBSs derived from ChIP-chip, and with their *in vitro* binding specificities available from at least one of the two PBM studies (27 unique TFs among 38 PWMs), we assigned PWM scores to all sites across the yeast genome, by moving the scoring window one base pair at a time. The PWM score assigned to each site represents the likelihood that the purified TF would bind to the site on naked DNA, and thus the intrinsic affinity between TF and DNA. That is, based on the gold standard consensus motif derived *in vitro*, the more similar the DNA sequence is to the consensus motif, the more likely the site on naked DNA is bound. As an example, Figure S2A shows the distributions of PWM scores of Leu3 across all possible binding sites throughout the genome (white), in the intergenic regions alone (grey), and in the *in vivo* TFBSs (green). Table S2 contains complete PWM score information for all other TFs. For these 27 TFs, we estimated the number of putative TFBSs in intergenic regions based on the lowest PWM score of the *in vivo* bound sites (red dotted line in Figure S2A. We term all sites above this threshold in intergenic regions "putative TFBSs" (Figure S2B, cyan).

Consistent with earlier estimates (Wasserman and Sandelin, 2004), our independent analysis shows that the proportions of the "*in vivo* TFBSs" (in all the growth conditions available) of the putative TFBSs are very small, with an average of 0.08% (± 0.05, SD) (Figure S2B, green) (see Table S2 for detailed information for all other TFs in this study). Since we employed the most stringent cut-offs, we expect the value of ~0.1% to be an upper bound to the estimate. Indeed, many binding sites that fit the consensus motif poorly are also thought to be bound by TFs (Tanay, 2006). Taking these weaker sites into account would

result in a larger number of putative TFBSs, and an even lower estimated fraction. When we considered the sites bound by TFs in YPD-grown yeast (termed “YPD-bound TFBSs”), this fraction decreased further approximately by a half to less than 0.05% of all predicted sites (Figure S2B, orange).

Correlating in vitro TF binding preferences to in vitro nucleosome binding preference

We quantitatively assessed the similarities between the intrinsic DNA-binding preference of TFs and nucleosomal histones by individually correlating the *in vitro* TF binding likelihoods (*i.e.* PWM scores) of all possible binding sequences in the entire yeast genome, to the genome-wide nucleosome occupancy profiles determined *in vitro* (Kaplan et al., 2009; Segal et al., 2006). The correlations were performed between all the 32-bp means of TF binding likelihoods obtained by scoring PWMs (Badis et al., 2008; Zhu et al., 2009) as explained above, and all the 32-bp means of intrinsic nucleosome occupancies across the genome.

We computed Pearson correlation coefficients between the predicted intrinsic binding likelihood profiles of all TFs and the two independent *in vitro* nucleosome occupancy profiles (Kaplan et al., 2009; Segal et al., 2006). Positive, negative, and modest correlation coefficients were observed. These correlation coefficients varied only slightly when we used the means of different window sizes used to average the PWM scores (*i.e.* 50-bp and 100-bp instead of 32-bp windows, see examples in Figure S3A and Figure S3B). Consequently, we concluded that the positive or negative correlation observed was not an artefact of averaging window size.

We also computed Spearman correlation coefficients, and found that nearly identical results were obtained (data not shown), most likely because a large number of data points (~400,000) were used. To further confirm the significance of the positive and negative correlations, we computed the expected correlation coefficients by randomising the nucleosome binding profiles across the yeast genome and keeping the TF binding likelihoods fixed. The expected correlations from 1,000 randomisation experiments were all very close to zero (*i.e.* no correlation).

The distributions of correlation coefficients were arbitrarily divided into three equal intervals (as shown in Figure 3 in the main text). We then categorised TFs in three groups, according to the correlation coefficients: (1) TFs that have a positive correlation with intrinsic nucleosome binding preference (histone-correlated, HC); (2) TFs that have a negative correlation (histone-anti-correlated, HA); and finally (3) TFs in the intermediate group that show a weak correlation or a disagreement between PBM and/or nucleosome binding preference publications. A positive correlation means a TF intrinsically prefers to bind to DNA sequences similar to the regions also preferred by histones on naked genomic DNA. A complete list of TFs and their Pearson correlation coefficients with respect to nucleosome binding preferences can be found in Table S1.

To explore the biological significance of this HC/HA classification, we related it to several TF properties including the DNA-binding domains (DBDs) the TFs possess, A/T proportion in genomic DNA sequences, and regulatory modes (activator/repressor), as described in the main text and in Table S3.

Identifying TF binding sites bound in YPD

From the total 4,387 highest confidence binding sites determined using ChIP-chip and conserved motif searches (MacIsaac et al., 2006), that is “*in vivo* TFBSs”, we identified the TFBSs which are bound in YPD (rich medium) based on the following two criteria. Firstly, the TFBS has to be within 700 bp upstream of the translation start site, where a proximal TFBS is likely to be located. Secondly, the *P*-value of intergenic probes in the ChIP-chip experiments of yeast grown in the YPD condition (Harbison et al., 2004), in which the binding site is located, has to be smaller than the most stringent cut-off of 0.001. Using these criteria, we identified 1,963 binding sites occupied by TFs under the YPD condition, which

we termed “YPD-bound TFBSs”. The TFBSs bound in galactose-supplemented medium were identified using the same criteria.

Estimating the fractions of binding sites within nucleosome-enriched regions
We superimposed the two *in vitro* genome-wide nucleosome occupancy profiles from (Kaplan et al., 2009; Segal et al., 2006) on the computationally predicted TFBSs (*i.e.* “putative TFBSs”, discovered by our computational exhaustive search explained above), the experimentally determined TFBSs (*i.e.* “*in vivo* TFBSs”), and the TFBSs bound in YPD (*i.e.* “YPD-bound TFBSs”). For the Segal *et al.* data set (Segal et al., 2006), we followed the original study that considered the sites with nucleosome occupancy greater than the cut-off of 0.5 (very stable nucleosomes) as the “nucleosome-enriched” (NE) sites, which are likely to be occluded by “stable” nucleosomes. In contrast, the sites in regions where nucleosome occupancy of less than 0.5, are considered to be “nucleosome-depleted” (ND) sites, which are likely to be nucleosome-free. This resulted in ~82% of the yeast genome within nucleosome-enriched regions, which is close to ~81%, the percentage of the genome covered by “well-positioned” and “fuzzy” nucleosomes, as defined by Lee and colleagues in their study (Lee et al., 2007), where nucleosome positions were determined in yeast grown in YPD.

For the Kaplan *et al. in vitro* data set (Kaplan et al., 2009), we identified the sites that have log-ratios between the number of reads that cover a particular base pair and the average across the genome above zero, *i.e.* nucleosome occupancy above genome-wide average, as nucleosome-enriched (NE) sites (and thus below zero as nucleosome-depleted (ND) sites). Using these criteria originally used by the authors, we estimated the fraction of the putative TFBSs that are likely to be occluded by nucleosomes (NE), based on the intrinsic binding sequence preferences. For the YPD-bound TFBSs, we also aligned their binding positions with genome-wide nucleosome occupancy profiles derived in the YPD growth condition (Kaplan et al., 2009; Lee et al., 2007), and computed the fraction of TFBSs within nucleosome-enriched regions. We then repeated this analysis for different groups of HC/HA TF classifications, and for different regulatory modes (activator/repressor/dual) (Figure 5 in main text and Figure S5). The expected numbers of TFBSs within the NE and ND regions were obtained by shuffling nucleosome occupancies over the YPD-bound sites over 1,000 experiments.

Non-intrinsic factors are at least as important as intrinsic nucleosome occupancy in determining a global framework of TF accessibility
We quantify the influence of intrinsic sequence specificity *versus* non-intrinsic factors by comparing TFBS coincidence with nucleosome occupancy under *in vitro* conditions *versus in vivo*, including YPD medium specifically. We first considered all the computed “putative TFBSs” in the intergenic regions, with PWM scores above the threshold as described earlier (Figure S2B, and Figure S5 top panel, cyan). We considered the sites with *in vitro* nucleosome occupancies (Kaplan et al., 2009) greater than the genome-wide average to be nucleosome-enriched (as explained above). Using this criterion, ~54% of putative TFBSs are located in nucleosome-enriched sites. That is, ~46% of putative TFBSs were predicted to be relatively accessible to TFs, which is less than the genome-wide average of ~50%

When we looked at the 4,387 “*in vivo* TFBSs” of 118 unique TFs, experimentally verified by ChIP-chip (Figure S2B and Figure S5 top panel, green), more than half (~54%) of TFBSs were predicted to be nucleosome-depleted based on these criteria. This result is consistent with an earlier study (Kaplan et al., 2009), which reported that the majority of bound TFBSs are nucleosome-depleted.

The difference between the fractions of accessible TFBSs in the putative TFBSs and *in vivo* TFBSs can be considered as the impact of intrinsic histone-DNA binding sequence preference on the outcome of TF binding events within a population of cells. The 8% (54% – 46%) difference is statistically significant (*P*-value ~$5x10^{-5}$, Welch’s *t*-test computed for the

binding sites of different TFs). This result supports the idea that stable nucleosomes help minimise the binding of TFs to non-functional sites. This is true even when different nucleosome data sets (Lee et al., 2007; Segal et al., 2006) are used. The proportions of *in vivo* TFBSs within nucleosome-enriched regions are also significantly smaller than those of the putative TFBSs, although the precise magnitude of the difference varies among the nucleosome profiles (Figure S4A-D and Figure S5 middle panel). Focusing only on the sites bound by TFs in the YPD growth condition (Figure S2B and Figure S5 top panel, orange), a similar fraction of TFBSs (~56%) was predicted to be nucleosome-depleted.

In order to compare TF and nucleosome occupancy under identical conditions *in vivo*, we switch from the nucleosome occupancy profile determined *in vitro* to the nucleosome profile obtained in the YPD condition from the same study (Kaplan et al., 2009). Only ~29% of these YPD-bound TFBSs were located in the *in vivo* nucleosome-enriched regions, and thus ~71% could be considered accessible by TFs (Figure S5 top panel, orange). The 15% (71% − 56%) difference between nucleosome-enriched YPD-bound TFBSs according to the *in vitro* nucleosome profile and the profile derived in YPD is statistically significant (*P*-value ~$2x10^{-12}$). This 15% relative difference (17% for the HC and 14% for the HA TFs, Figure 5 in main text) can be considered as the influence of non-intrinsic factors such as *in vivo* TF binding and the recruitment of histone-modifying enzymes, and chromatin remodellers. This combined non-intrinsic effect of about 15% is markedly greater than the effect of intrinsic histone-DNA binding preference on TF binding (*c.f.* 8% in this study).

Our finding from this comprehensive TF set therefore supports earlier studies showing that non-intrinsic factors play an important role in determining the binding configurations of histones and TFs *in vivo* (Koerber et al., 2009; Owen-Hughes and Workman, 1994; Zhang et al., 2009). Notably, the 29% of sites bound simultaneously by TFs and histones (Figure S5 top panel, orange) might be utilised by the so-called pioneer TFs that can bind nucleosomes stably (Sekiya et al., 2009).

To avoid uncertainty due to the threshold for nucleosome-enrichment, we repeated this analysis using different intrinsic and YPD nucleosome occupancy profiles (Lee et al., 2007; Segal et al., 2006) (Figure S5 middle panel). With these alternative data sets, we obtained consistent results revealing that the non-intrinsic factors, such as the interplay between different DNA-binding proteins, are at least as important to the *in vivo* TF accessibility as the intrinsic sequence preference alone.

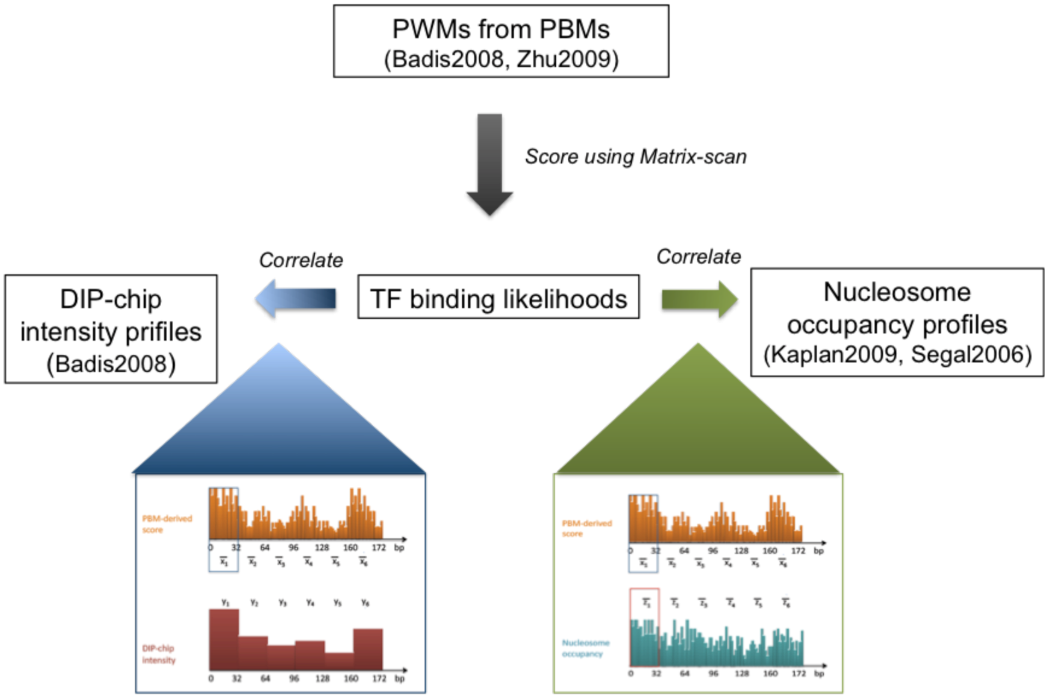

Figure S1. Summary of TF Binding Likelihood Assignment and Correlating with Nucleosome Occupancy Profiles, Related to Figure 1

Flowchart summarising the TF binding likelihood assignment, and nucleosome binding preference correlation. The PWMs obtained from the *in vitro* PBM experiments (Badis et al., 2008; Zhu et al., 2009) were scored against the entire yeast genome, and cross-validated with the highly enriched TF-bound sequences obtained from the DIP-chip experiment at 32-bp resolution (Badis et al. 2008). The genome-wide intrinsic binding likelihood profile of each TF was then correlated with genome-wide nucleosome occupancy profiles determined *in vitro*. The correlation was also performed between the mean intrinsic TF binding likelihoods (*i.e.* PWM scores) and nucleosome occupancies (Segal et al. 2006; Kaplan et al. 2009) of all 32-bp windows across the genome. These correlations were used to classify TFs into histone-correlated (HC), histone-anti-correlated (HA), and intermediate (I) groups, based on similarity of their intrinsic DNA-binding specificities, compared with those of nucleosomal histones.

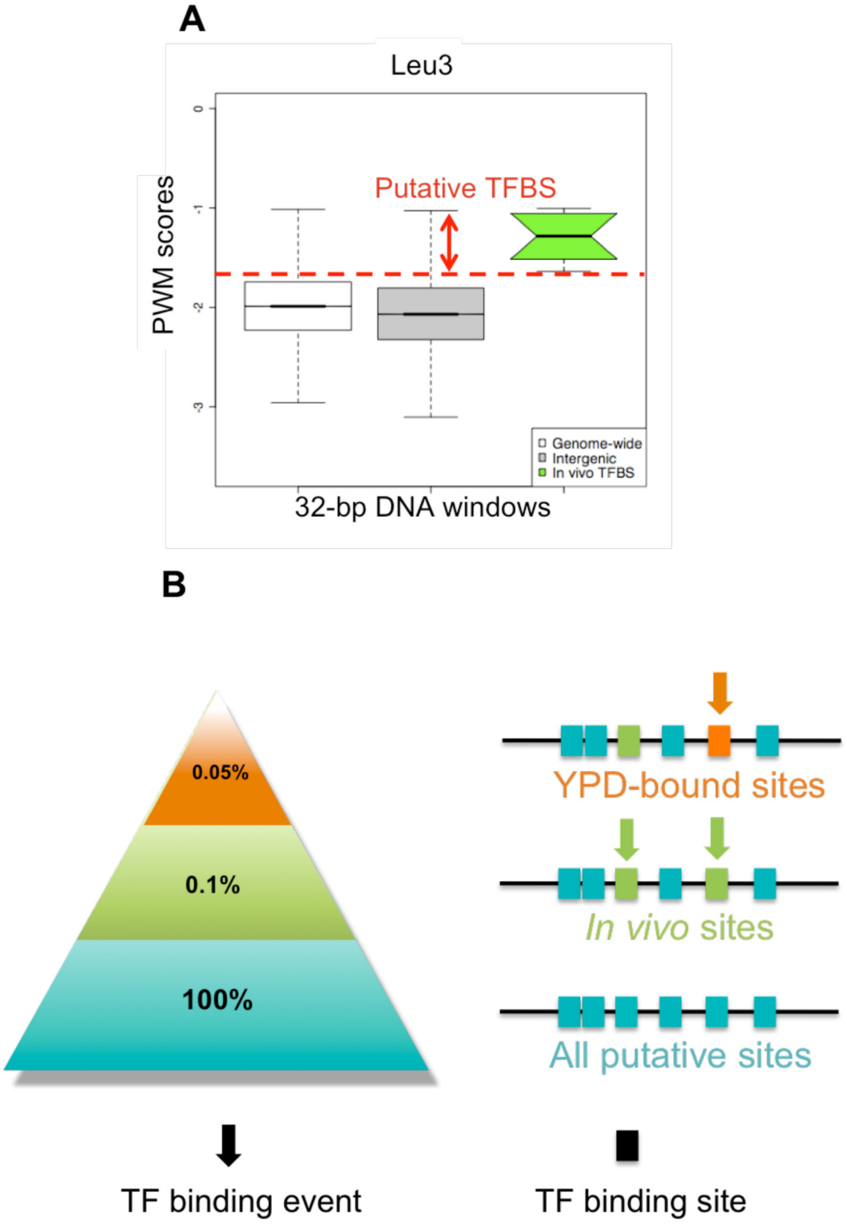

Figure S2. Summary of Number of TF Binding Sites, Related to Figure 2

(A) The distributions of PWM scores across all regions in the yeast genome (white box), in all intergenic regions (grey), and in experimentally determined *in vivo* binding sites (green). Shown here are boxplots of the PWM score distributions of Leu3, obtained from scoring the PWM from (Badis et al., 2008). The dotted red line indicates the minimal PWM scores of *in vivo* binding sites, which was used as a cut-off for searching for other putative binding sites in the intergenic regions. Notches were added to the boxes to indicate the differences between the boxes (strong evidence that the two medians significantly differ if the notches do not overlap). (B) Fractions of putative sites that are bound by TFs *in vivo* and in the YPD growth condition. We estimated that ~0.1% of computational putative TFBSs are bound *in vivo* and only ~0.05% in the YPD condition.

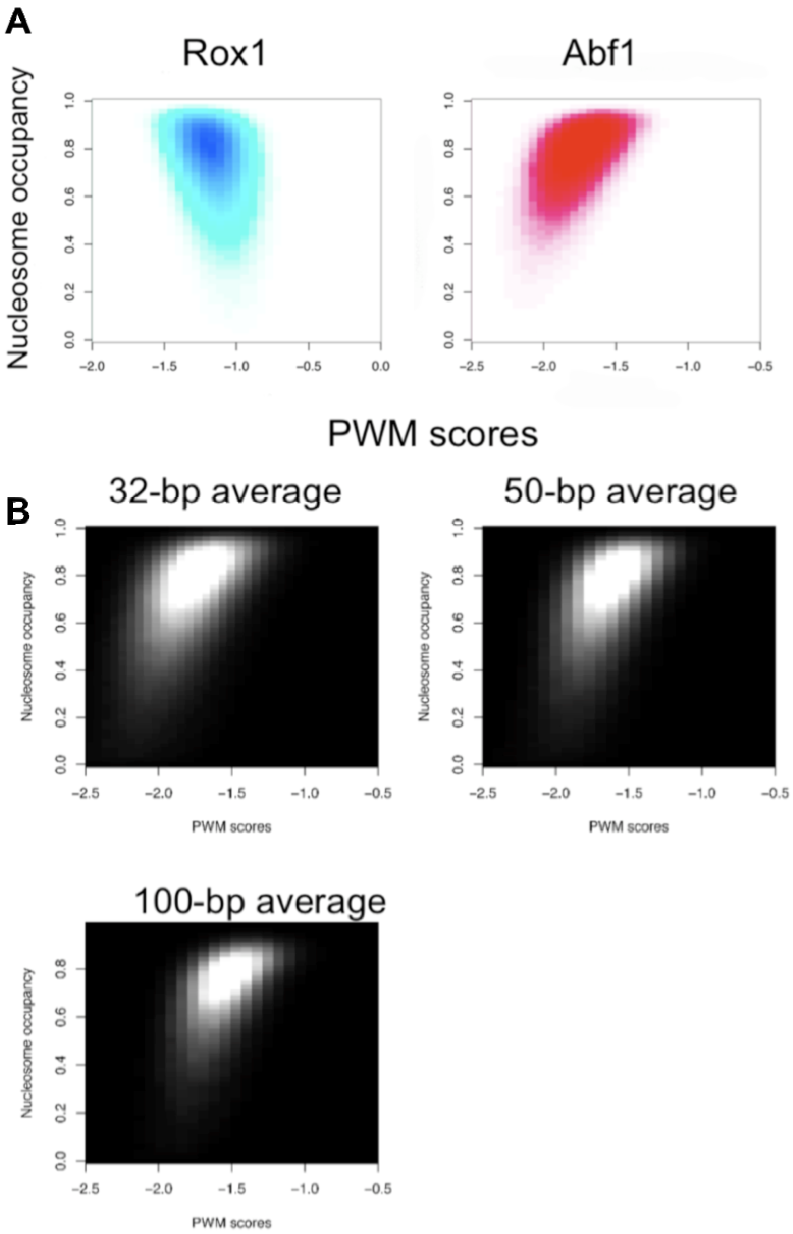

Figure S3. Heatmaps of Nucleosome Occupancy *versus* PWM Scores, Related to Figure 3

(A) Heatmaps correlating on the x-axis, the genome-wide TF binding likelihoods of Rox1 and Abf1, against the intrinsic nucleosome occupancy profiles on the y-axis. Rox1 has intrinsic DNA-binding specificities negatively correlated with that of histones (histone-anti-correlated, HA); whereas Abf1 shows a positive correlation with histones (histone-correlated, HC) (B) Heatmaps correlating the genome-wide TF binding likelihoods of Abf1, a histone-correlated (HC) TF, on the x-axis, against the intrinsic nucleosome occupancy profiles on the y-axis, using different window sizes (32 bp, 50 bp, 100 bp). The Pearson correlation coefficients between the two variables are 0.53 for all three window sizes.

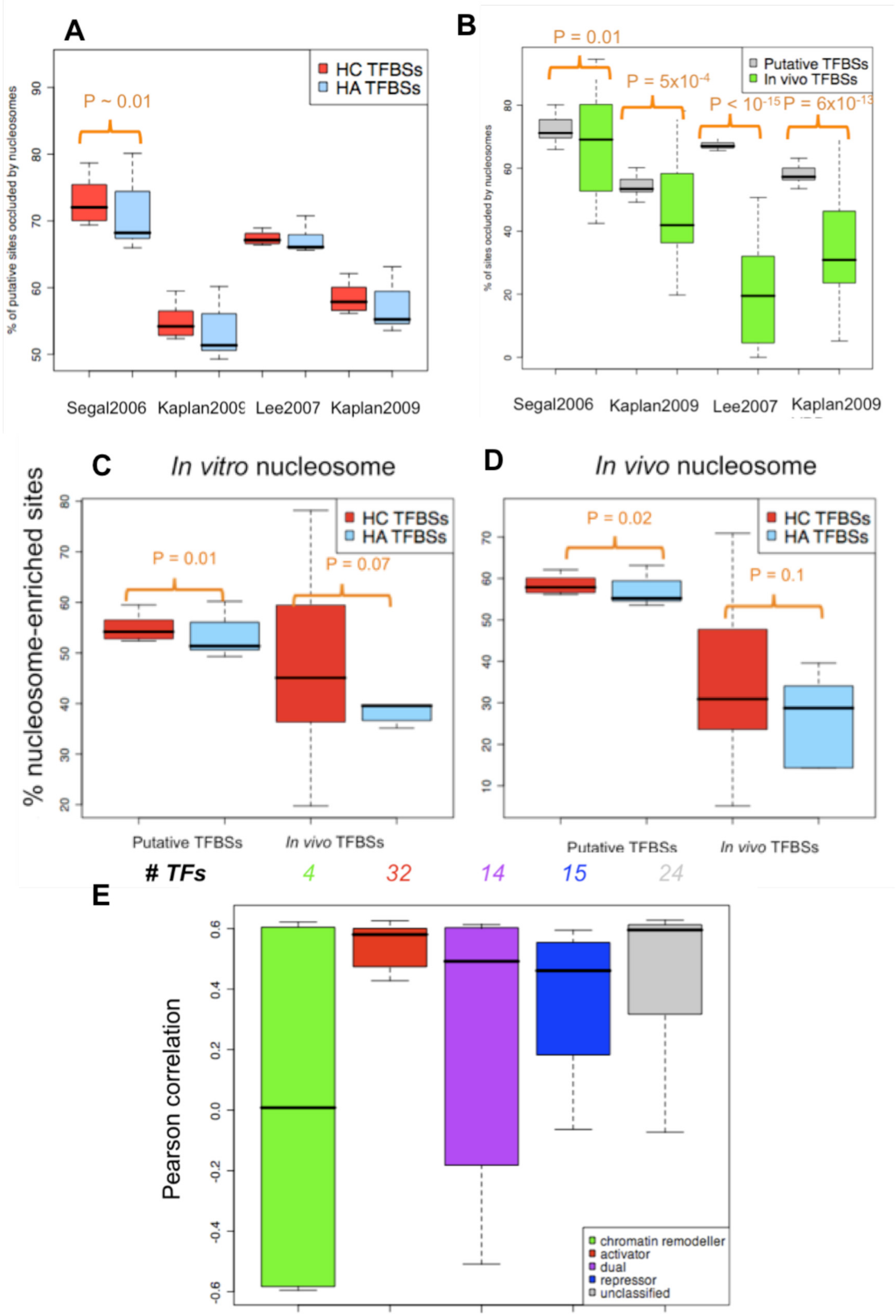
A
B
C
D
E
HC TFBSs
HA TFBSs
P ~ 0.01
% of putative sites occluded by nucleosomes
Segal2006
Kaplan2009
Lee2007
Kaplan2009
P = 0.01
P = 5x10-4
P < 10-15
P = 6x10-13
Putative TFBSs
In vivo TFBSs
% of sites occluded by nucleosomes
In vitro nucleosome
In vivo nucleosome
% nucleosome-enriched sites
P = 0.07
P = 0.02
P = 0.1
Putative TFBSs
In vivo TFBSs
TFs
4
32
14
15
24
Pearson correlation
chromatin remodeller
activator
dual
repressor
unclassified

Figure S4. Percentages of All Putative TFBSs within Nucleosome-Enriched (NE) Regions, Related to Figure 4

(A) Percentages of all putative TFBSs within nucleosome-enriched regions (as defined in the main text), shown separately for four different genome-wide nucleosome profiles, and for the TFBSs bound by different histone-correlated, HC, and histone-anti-correlated, HA, TFs. A significantly greater fraction of putative TFBSs bound by the HC TFs (shown in red) are more nucleosome-enriched than the HA TFBSs (shown in blue). *P*-values for HC being greater are approximately 0.01 for all nucleosome profiles (Mann-Whitney).

(B) Percentages of *in vivo* TFBSs and all putative TFBSs likely to be occluded by nucleosomes, shown separately for four different nucleosome occupancy profiles as described before. The percentages of *in vivo* TFBSs occluded by nucleosomes are lower than of putative TFBSs in all the nucleosome profiles. *P*-values of putative TFBSs locating within nucleosome-enriched regions more frequently than *in vivo* TFBSs (Mann-Whitney) are shown in orange. The ranges in the *in vivo* nucleosome profiles are greater than the *in vitro* nucleosome profiles.

(C) The percentages of TF binding sites within *in vitro* nucleosome-enriched regions (*i.e.* occluded by *in vitro* nucleosomes) (Kaplan et al., 2009). As expected, predicted binding sites ("putative TFBSs") of the HC TFs are more likely to be occluded by nucleosomes, compared to those of HA TFs. Similarly, experimentally verified binding sites ("*in vivo* TFBSs") of the histone-correlated, HC, TFs are likely to be occluded more often than those of the histone-anti-correlated, HA, TFs, with less statistical significance. This is likely to be due to the influence of the competition between TFs and histones to bind to DNA sequence in cells. *P*-values were computed using the Mann-Whitney test.

(D) The percentages of TF binding sites occluded by *in vivo* nucleosomes (Kaplan et al., 2009). Both putative and *in vivo* TFBSs of the HC TFs are more likely to be occluded by nucleosomes, compared to those of the HA group, despite less significant statistics. However, we noted that the *in vivo* TFBSs and *in vivo* nucleosome occupancy (the last two boxplots) are interdependent and thus do not reflect their intrinsic binding preferences to DNA sequences in the same way the *in vitro* TFBSs and *in vitro* nucleosome occupancy (the first two boxplots in C). In summary, we have shown that the TFBSs of the HC TFs overlap with the sites bound by histones more often than those of the HA TFs, regardless of the type of TF binding data (binding preference landscape of a whole genome (as used in the main text), computationally predicted, and *in vivo* TFBSs (as shown above)), or neither nucleosome binding profile data sets (*in vitro* or *in vivo*).

(E) Boxplots of the Pearson correlation coefficients of genome-wide intrinsic DNA-binding profiles TFs and nucleosomes, plotted separately for TFs with different regulatory functions. Comparison between binding preference of TFs from (Zhu et al. 2009) data set against the nucleosome occupancy profile from (Kaplan et al. 2008) data set is demonstrated here. The average correlations of activators are significantly higher than of repressors (Mann-Whitney *P*-value ~0.005).

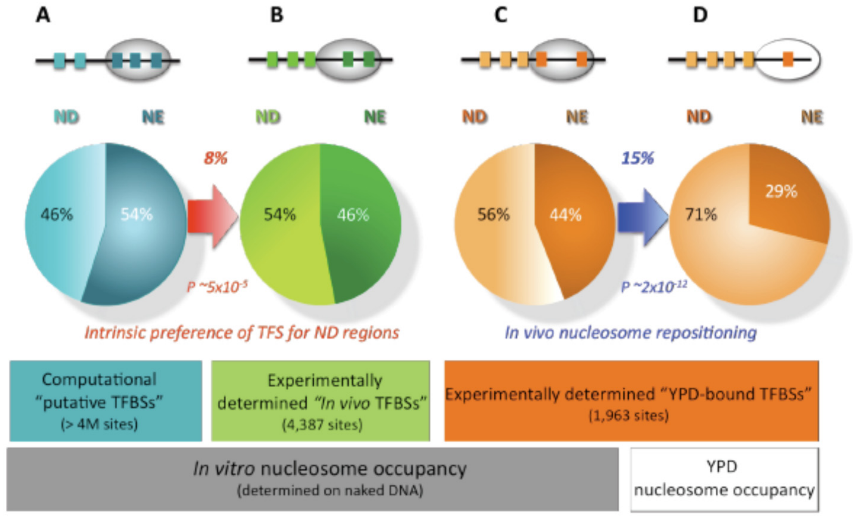

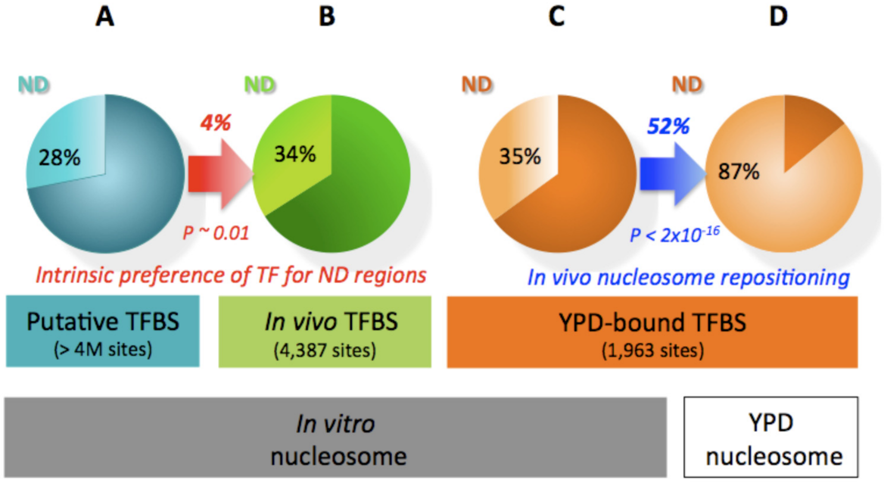

Intermediate and unclassified TFBSs

Dual and other TFBSs

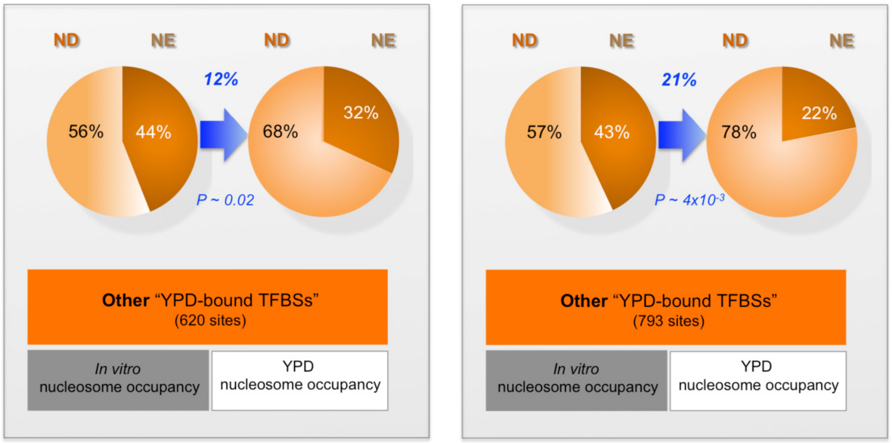

Figure S5. The Proportions of Nucleosome-Enriched (NE) and Nucleosome-Depleted (ND) TFBSs of Different Types and with Different Nucleosome Occupancy Profiles, Related to Figure 5

(Upper panel) Nucleosome-bound proportions are shown in the darker shades. (A) Forty-six percent of all computationally predicted sites in the intergenic regions fall into the regions that have nucleosome occupancy lower than average (Kaplan et al., 2009) (arbitrarily considered as nucleosome-deplete regions). (B) The TFBSs bound by different TFs *in vivo* have a significantly greater percentage of accessible sites and smaller percentage of sites in the NE regions (*P*-value ~$5x10^{-5}$, Welch's *t*-test). This difference exemplifies the role of global nucleosome positioning that restricts the accessibility of many putative sites. (C) A similar fraction of the sites bound in the YPD conditions is predicted to be in ND, compared to in the *in vivo* bound binding sites. (D) A significantly larger fraction of these sites are accessible (in ND) when their positions were aligned to condition-specific (YPD) nucleosome occupancy from the same study (Kaplan et al., 2009) (*P*-value ~$2x10^{-12}$). This suggests an intricate nucleosome repositioning occurs *in vivo*.

(Middle panel) (A) Only ~28% of all the putative sites in the intergenic regions are predicted to be nucleosome-depleted. While the *in vivo* binding sites (B), obtained from (MacIsaac et al. 2006), have greater percentage of accessible sites and smaller percentage of nucleosome-enriched sites. In other words, the global nucleosome occupancy restricts the accessibility of the majority of the putative sites. The "stable" nucleosome occupancy is predicted from the probabilistic model of nucleosome binding preference (Segal et al. 2006). (C) A very similar fraction of the sites bound in the YPD condition is predicted to be nucleosome-depleted, compared to the *in vivo* bound sites. (D) A significantly larger fraction of these sites become accessible when their positions were aligned to condition-specific (YPD) nucleosome positions (Lee et al. 2007). This suggests an intricate nucleosome repositioning/disrupting occurs at TFBSs *in vivo*. Note that genome-wide intergenic regions are ~32% nucleosome-depleted.

(Lower panel) The proportions of nucleosome-enriched (NE) and nucleosome-depleted (ND) TFBSs of the HC and HA TF groups, based on *in vitro* and *in vivo* nucleosome occupancy profiles (Kaplan *et al.* 2009). This is the same analysis as seen in Figure 5 in the main text but performed separately for the intermediate and unclassified TFBSs, *i.e.* neither HC nor HA (left), and for dual and other regulatory mode TFBSs, *i.e.* neither activator nor repressor (right).

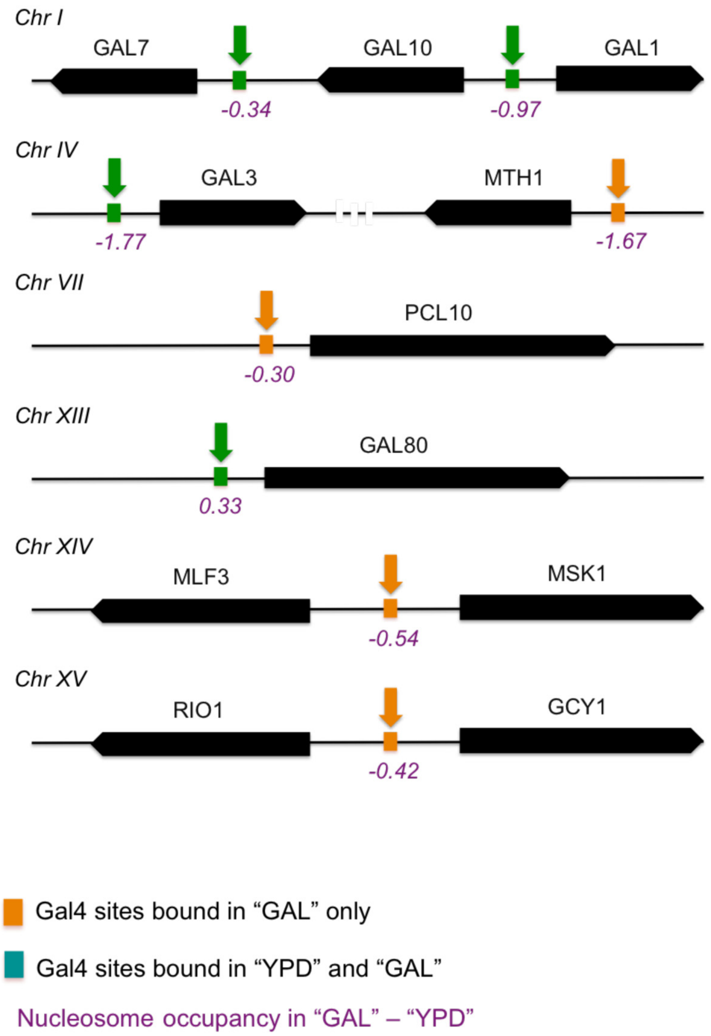

Figure S6. Dynamics of Nucleosome and Gal4 Binding in Yeast Grown in YPD (Rich) and GAL (Galactose-Supplemented) Media, Related to Figure 6

Out of eight Gal4 TFBSs experimentally determined (eleven target genes), four are bound by TFs in both conditions (green) and the other four are bound only in the GAL medium (orange). Nucleosome occupancies (log-ratios over genome-wide average) in 32-bp windows around the Gal4 TFBSs of yeast grown under the GAL condition are consistently lower than those in YPD, except for the one upstream of GAL80. This suggests that nucleosomes are repositioned upon Gal4 binding, allowing more Gal4 molecules and potentially other TFs to access the cognate sites and activate Gal4 target genes, which are necessary for galactose metabolism.

Supplemental References

Badis, G., Chan, E.T., van Bakel, H., Pena-Castillo, L., Tillo, D., Tsui, K., Carlson, C.D., Gossett, A.J., Hasinoff, M.J., Warren, C.L., *et al.* (2008). A library of yeast transcription factor motifs reveals a widespread function for Rsc3 in targeting nucleosome exclusion at promoters. Mol Cell *32*, 878-887.

Berger, M.F., Philippakis, A.A., Qureshi, A.M., He, F.S., Estep, P.W., 3rd, and Bulyk, M.L. (2006). Compact, universal DNA microarrays to comprehensively determine transcription-factor binding site specificities. Nat Biotechnol *24*, 1429-1435.

Dwight, S.S., Harris, M.A., Dolinski, K., Ball, C.A., Binkley, G., Christie, K.R., Fisk, D.G., Issel-Tarver, L., Schroeder, M., Sherlock, G., *et al.* (2002). Saccharomyces Genome Database (SGD) provides secondary gene annotation using the Gene Ontology (GO). Nucleic Acids Res *30*, 69-72.

Gordan, R., Hartemink, A.J., and Bulyk, M.L. (2009). Distinguishing direct versus indirect transcription factor-DNA interactions. Genome Res *19*, 2090-2100.

Gordan, R., Murphy, K.F., McCord, R.P., Zhu, C., Vedenko, A., and Bulyk, M.L. (2011). Curated collection of yeast transcription factor DNA binding specificity data reveals novel structural and gene regulatory insights. Genome Biol *12*, R125.

Harbison, C.T., Gordon, D.B., Lee, T.I., Rinaldi, N.J., Macisaac, K.D., Danford, T.W., Hannett, N.M., Tagne, J.B., Reynolds, D.B., Yoo, J., *et al.* (2004). Transcriptional regulatory code of a eukaryotic genome. Nature *431*, 99-104.

Hertz, G.Z., and Stormo, G.D. (1999). Identifying DNA and protein patterns with statistically significant alignments of multiple sequences. Bioinformatics *15*, 563-577.

Kaplan, N., Moore, I.K., Fondufe-Mittendorf, Y., Gossett, A.J., Tillo, D., Field, Y., LeProust, E.M., Hughes, T.R., Lieb, J.D., Widom, J., and Segal, E. (2009). The DNA-encoded nucleosome organization of a eukaryotic genome. Nature *458*, 362-366.

Koerber, R.T., Rhee, H.S., Jiang, C., and Pugh, B.F. (2009). Interaction of transcriptional regulators with specific nucleosomes across the Saccharomyces genome. Mol Cell *35*, 889-902.

Lee, W., Tillo, D., Bray, N., Morse, R.H., Davis, R.W., Hughes, T.R., and Nislow, C. (2007). A high-resolution atlas of nucleosome occupancy in yeast. Nat Genet *39*, 1235-1244.

Liu, X., Noll, D.M., Lieb, J.D., and Clarke, N.D. (2005). DIP-chip: rapid and accurate determination of DNA-binding specificity. Genome Res *15*, 421-427.

MacIsaac, K.D., Wang, T., Gordon, D.B., Gifford, D.K., Stormo, G.D., and Fraenkel, E. (2006). An improved map of conserved regulatory sites for Saccharomyces cerevisiae. BMC Bioinformatics *7*, 113.

Owen-Hughes, T., and Workman, J.L. (1994). Experimental analysis of chromatin function in transcription control. Crit Rev Eukaryot Gene Expr *4*, 403-441.

Segal, E., Fondufe-Mittendorf, Y., Chen, L., Thastrom, A., Field, Y., Moore, I.K., Wang, J.P., and Widom, J. (2006). A genomic code for nucleosome positioning. Nature *442*, 772-778.

Sekiya, T., Muthurajan, U.M., Luger, K., Tulin, A.V., and Zaret, K.S. (2009). Nucleosome-binding affinity as a primary determinant of the nuclear mobility of the pioneer transcription factor FoxA. Genes Dev *23*, 804-809.

Sharon, E., Kalma, Y., Sharp, A., Raveh-Sadka, T., Levo, M., Zeevi, D., Keren, L., Yakhini, Z., Weinberger, A., and Segal, E. (2012). Inferring gene regulatory logic from high-

throughput measurements of thousands of systematically designed promoters. Nat Biotechnol. *in press.*

Tanay, A. (2006). Extensive low-affinity transcriptional interactions in the yeast genome. Genome Res *16*, 962-972.

Thomas-Chollier, M., Sand, O., Turatsinze, J.V., Janky, R., Defrance, M., Vervisch, E., Brohee, S., and van Helden, J. (2008). RSAT: regulatory sequence analysis tools. Nucleic Acids Res *36*, W119-127.

Wasserman, W.W., and Sandelin, A. (2004). Applied bioinformatics for the identification of regulatory elements. Nat Rev Genet *5*, 276-287.

Wilson, D., Charoensawan, V., Kummerfeld, S.K., and Teichmann, S.A. (2008). DBD--taxonomically broad transcription factor predictions: new content and functionality. Nucleic Acids Res *36*, D88-92.

Zhang, Y., Moqtaderi, Z., Rattner, B.P., Euskirchen, G., Snyder, M., Kadonaga, J.T., Liu, X.S., and Struhl, K. (2009). Intrinsic histone-DNA interactions are not the major determinant of nucleosome positions in vivo. Nat Struct Mol Biol *16*, 847-852.

Zhu, C., Byers, K.J., McCord, R.P., Shi, Z., Berger, M.F., Newburger, D.E., Saulrieta, K., Smith, Z., Shah, M.V., Radhakrishnan, M.*, et al.* (2009). High-resolution DNA-binding specificity analysis of yeast transcription factors. Genome Res *19*, 556-566.
